# Motion‐corrected and high‐resolution anatomically assisted (MOCHA) reconstruction of arterial spin labeling MRI

**DOI:** 10.1002/mrm.28205

**Published:** 2020-03-03

**Authors:** Abolfazl Mehranian, Colm J. McGinnity, Radhouene Neji, Claudia Prieto, Alexander Hammers, Enrico De Vita, Andrew J. Reader

**Affiliations:** ^1^ Department of Biomedical Engineering School of Biomedical Engineering and Imaging Sciences King’s College London London United Kingdom; ^2^ School of Biomedical Engineering and Imaging Sciences, King’s College London and King’s College London & Guy’s and St. Thomas’ PET Centre, St. Thomas’ Hospital London United Kingdom; ^3^ MR Research Collaborations Siemens Healthcare Frimley United Kingdom

**Keywords:** anatomical priors, arterial spin labeling, partial‐volume correction, perfusion MRI, reconstruction

## Abstract

**Purpose:**

A model‐based reconstruction framework is proposed for motion‐corrected and high‐resolution anatomically assisted (MOCHA) reconstruction of arterial spin labeling (ASL) data. In this framework, all low‐resolution ASL control‐label pairs are used to reconstruct a single high‐resolution cerebral blood flow (CBF) map, corrected for rigid‐motion, point‐spread‐function blurring and partial volume effect.

**Methods:**

Six volunteers were recruited for CBF imaging using pseudo‐continuous ASL labeling, two‐shot 3D gradient and spin‐echo sequences and high‐resolution T_1_‐weighted MRI. For 2 volunteers, high‐resolution scans with double and triple resolution in the partition direction were additionally collected. Simulations were designed for evaluations against a high‐resolution ground‐truth CBF map, including a simulated hyperperfused lesion and hyperperfusion/hypoperfusion abnormalities. The MOCHA technique was compared with standard reconstruction and a 3D linear regression partial‐volume effect correction method and was further evaluated for acquisitions with reduced control‐label pairs and k‐space undersampling.

**Results:**

The MOCHA reconstructions of low‐resolution ASL data showed enhanced image quality, particularly in the partition direction. In simulations, both MOCHA and 3D linear regression provided more accurate CBF maps than the standard reconstruction; however, MOCHA resulted in the lowest errors and well delineated the abnormalities. The MOCHA reconstruction of standard‐resolution in vivo data showed good agreement with higher‐resolution scans requiring 4‐times and 9‐times longer acquisitions. The MOCHA reconstruction was found to be robust for 4‐times‐accelerated ASL acquisitions, achieved by reduced control‐label pairs or k‐space undersampling.

**Conclusion:**

The MOCHA reconstruction reduces partial‐volume effect by direct reconstruction of CBF maps in the high‐resolution space of the corresponding anatomical image, incorporating motion correction and point spread function modeling. Following further evaluation, MOCHA should promote the clinical application of ASL.

## 1 INTRODUCTION

Arterial spin labeling (ASL) is a noninvasive perfusion‐weighted MRI technique for the quantification of cerebral blood flow (CBF),^1^ using magnetically labeled blood water as an endogenous contrast agent. In this technique, blood spins are typically labeled by inversion before flowing into the imaging volume, with pseudo‐continuous ASL (pCASL) currently as the preferred method.^1^ The difference between label and control (ie, non‐labeled) images produces a signal proportional to the local tissue blood flow.^2^ Arterial spin labeling has an intrinsically low SNR, as the volume of labeled blood is only about 1%‐2% of total cerebral blood volume (about 4%‐5%), and the magnetic label decays by the T_1_ relaxation time of blood, while it flows from the labeling region to imaging volume. To allow the labeled blood to reach the imaging volume, the ASL signal is acquired following a post‐label delay (PLD) time. Short PLDs are associated with less T_1_ decay and higher SNR; however, too short PLDs may be insufficient for full arrival of labeled blood into the tissues, leading to inaccurate CBF quantification.

To improve SNR, typically 10‐50 control‐label (C‐L) pairs with low nominal spatial resolution (in‐plane: 3‐4 mm, through‐plane: 4‐8 mm) are acquired and averaged.^1^ In addition, background suppression,^3^ 3D readout sequences,^4^ and parallel imaging^5^ are also used to respectively suppress static tissues, increase the SNR and brain coverage, and reduce acquisition time. Although reducing spatial resolution improves SNR, it results in partial‐volume averaging of gray‐matter (GM) and white‐matter (WM) CBF.^6^ Moreover, the widely used 3D readout sequences such as gradient and spin echo (GRASE)^7^ can introduce substantial through‐plane blurring (due to the T_2_ decay of signal across echo trains) and hence contribute to partial‐volume effects (PVE). For single‐shot GRASE, the through‐plane point spread function (PSF) has been reported to be from 1.5 to 1.9 voxels (FWHM).^8^ Segmented acquisition schemes help minimize this effect; however, as the number of shots increases, the acquisition time and sensitivity to motion also increase.^9^

For partial‐volume correction (PVC), existing methods aim to unmix GM and WM signals (overlapping in low‐resolution acquisitions) using partial‐volume (PV) estimates obtained from anatomical MR images. They are linear regression (LR),^10^ modified least trimmed squares,^11^ or Bayesian inference for ASL.^6^ Partial‐volume estimation requires accurate registration, segmentation, and downsampling of the anatomical MR images into the ASL image resolution, which are prone to errors.^12^ These PVC methods can be preceded by a deconvolution preprocessing step to reduce the PSF blurring^9^; however, deconvolution is known to amplify noise and can result in Gibbs ringing artifacts. Partial‐volume effects can be reduced by increasing the acquisition’s spatial resolution; however, the reduced SNR requires more averaging (ie, longer acquisition time), which increases motion sensitivity. Hence, a number of denoising^13^ and undersampled MRI techniques^14^ have been proposed to reduce noise while using as few averages as possible. Currently, PVC involves several preprocessing steps of ASL images (deconvolution, denoising, and motion correction) and of structural MR image (registration, segmentation, and downsampling).^15^ The actual PVC step is then typically carried out in the image space of the low‐resolution C‐L pairs, whereas operating at a higher resolution might improve their performance.^16^

In this study, we propose a framework for reconstruction of low‐resolution ASL data into the high‐resolution space of the anatomical images, corrected for motion, PSF blurring, and undersampling artifacts, with additional noise reduction. To effectively reduce noise and PVE, first, all C‐L pairs are simultaneously used to reconstruct a single perfusion‐weighted ASL image compared with the standard methods in which the C‐L data are separately reconstructed, motion‐corrected, subtracted, and then averaged. Second, a smoothness prior, weighted by the anatomical image, is used to assist the reconstruction of the target high‐resolution perfusion image. In this work, the proposed motion‐corrected and high‐resolution anatomically assisted (MOCHA) ASL image reconstruction method was evaluated using simulations and in vivo data sets and compared with the standard reconstruction methods and a 3D LR (3DLR) method.^17^

## 2 METHODS

### 2.1 MOCHA reconstruction

**Figure 1:**
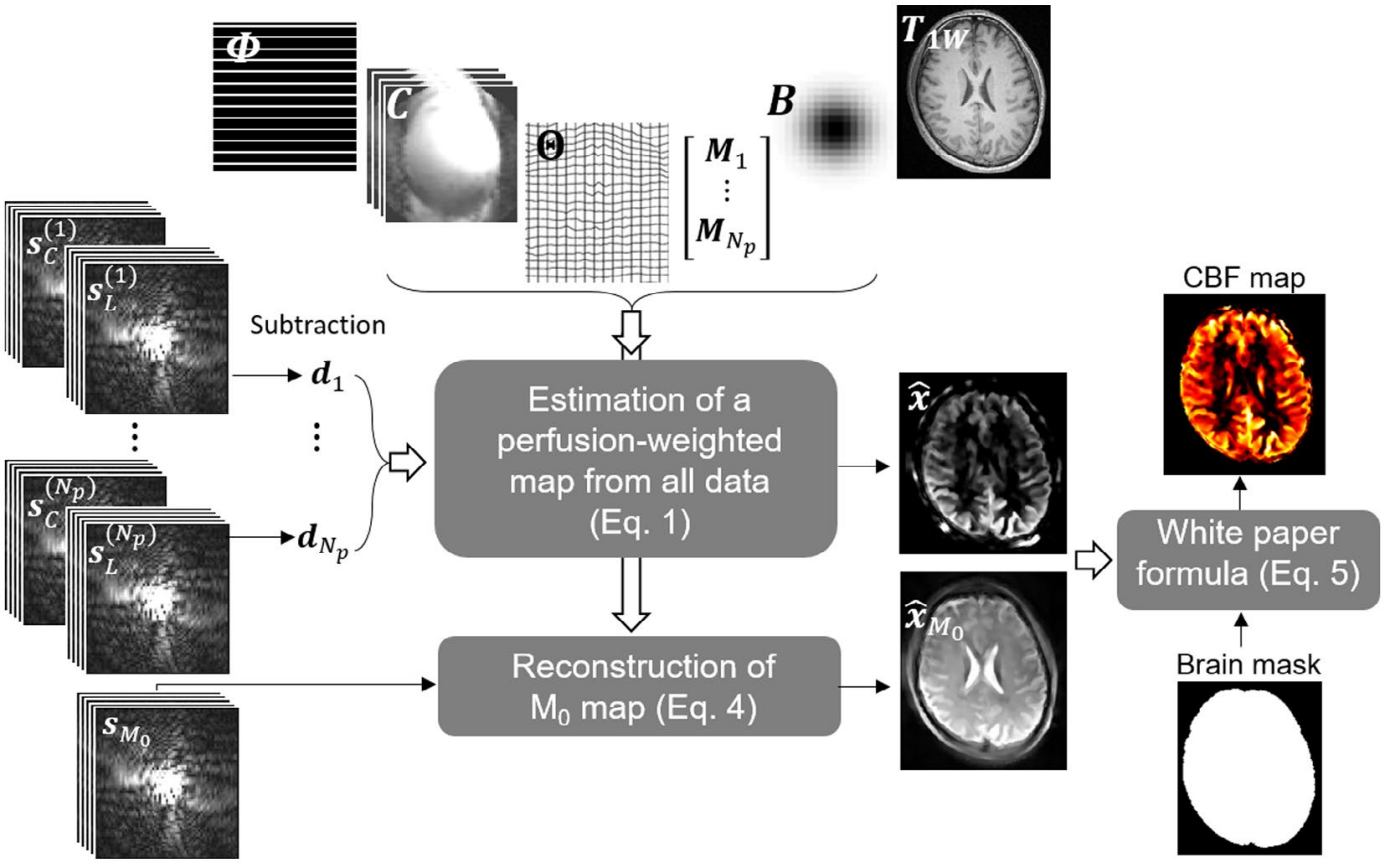
Flowchart of the motion‐corrected and high‐resolution anatomically assisted (MOCHA) reconstruction algorithm

Reconstruction of a high‐resolution perfusion‐weighted image, $x\in C^{N_{h}}$, from $N_{p}$ pairs of low‐resolution C‐L ASL data was formulated as the following model‐based minimization problem^18^:

(1) $$\hat{x}=\underset{x}{\text{argmin}}\left\{\frac{1}{2N_{p}}\sum_{i}^{N_{p}}{∥{\mathrm{ET}}_{i}\mathrm{Bx}-d_{i}∥}_{W}^{2}+\beta R\left(x\right)\right\}$$

where $d_{i}\in C^{N_{m}L}$ is the element‐wise subtraction of the *i*th control and the labeled multichannel k‐space data (ie, perfusion‐weighted data), $N_{m}$ and L are the number of k‐space samples and the number of coils. $B\in R^{N_{h}\times N_{h}}$ is a convolution operator used to model PSF blurring of the MR sequence in image space, where $N_{h}$ is the number of voxels in the high‐resolution MR image. $T_{i}=\Theta {\mathrm{DM}}_{i}\in R^{N_{l}\times N_{h}}$ consists of the rigid transformation of x to the *i*th motion state ( $M_{i}$) (see Section 2.3.1), downsampling ( D) to ASL low‐resolution space and non‐rigid geometric distortion ( Θ) induced by B_0_ field inhomogeneity, which was set to identity in this work. $N_{l}$ is the number of voxels in the ASL space. $E=\left(I_{L}⊗\Phi F\right)C\in C^{N_{m}L\times N_{l}}$ consists of a coil sensitivity matrix of L coils ( $C\in C^{N_{l}L\times N_{l}}$), Fourier transform ( $F\in C^{N_{l}\times N_{l}}$) and k‐space undersampling matrix ( $\Phi \in R^{N_{m}\times N_{l}}$) with $N_{m}\leq N_{l}$ samples and ⊗ representing the Kronecker product and $I_{L}$ representing the identity matrix of size L. $W\in R^{N_{m}L\times N_{m}L}$ is the weighting matrix obtained from the inversion of the noise covariance matrix,^19^ which was set to identity in this work. Figure 1 provides a flowchart describing the forward model used in Equation 1. R(x) is a penalty function defined as a weighted quadratic prior as follows:

(2) $$R\left(x\right)=\sum_{j}^{N_{h}}\sum_{b\in N_{j}}\omega_{\mathrm{jb}}\xi_{\mathrm{jb}}{\left(x_{j}-x_{b}\right)}^{2},$$

which aims to suppress noise and artifacts based on the intensity differences between voxels j and b in the neighborhood $N_{j}$, while preserving boundaries using the similarity coefficients $\omega_{\mathrm{jb}}$, calculated from the anatomical image. $\xi_{\mathrm{jb}}$ are proximity coefficients used to modulate the intensity differences based on their Euclidian distance. The β in Equation 1 is a regularization parameter. In this study, the similarity coefficients were defined using Gaussian kernels^20^ as follows:

(3) $$\omega_{\mathrm{jb}}=\frac{1}{\sqrt{2\pi }\sigma }\exp\left(-\frac{{\left(v_{j}-v_{b}\right)}^{2}}{2\sigma^{2}}\right)$$

where $v\in R^{N_{h}}$ is the MR anatomical image and σ is a shape hyperparameter. The reconstruction method in Equation 1 aims to perform PVC using a higher‐resolution image grid and PSF modeling. As described in Section 2.3.2, for CBF quantification, a calibration image (*M*_0_) is also acquired during the ASL scan. The *M*_0_ images were reconstructed using a method similar to Equation 1 but devised for individual k‐spaces as follows:

(4) $${\hat{x}}_{M_{0}}=\underset{x_{M_{0}}}{\text{argmin}}\left\{\frac{1}{2}{∥{\mathrm{ET}}_{M_{0}}{\mathrm{Bx}}_{M_{0}}-s_{M_{0}}∥}_{W}^{2}+\beta R\left(x_{M_{0}}\right)\right\}$$

where $s_{M_{0}}$ is the k‐space data of the $M_{0}$ data set and R is the same as defined in Equation 2. In this work, Equations 1 and 4 were solved using the steepest decent algorithm (see Appendix A).

### 2.2 In vivo data acquisition

Six healthy volunteers (all males, mean age (±SD) 40.1 ± 5.4 years) were scanned on a Siemens 3T Biograph PET‐MR scanner with a 12‐channel head coil. For perfusion imaging, a pCASL labeling scheme^21^ was used with a center‐out 3D‐GRASE readout with the following parameters: TR = 4000 ms, TE = 17.62 ms, flip angle = 150° (chosen to reduce blurring in the partition direction), image matrix = 64 × 62 × 29, nominal resolution = 4 × 4 × 4 mm^3^, reconstruction FOV = 256 × 256 × 104 mm^3^, slice oversampling = 10%, turbo factor = 29, EPI factor = 31, number of shots (segments) = 2, bandwidth = 3126 Hz/pixel, background suppression = on, labeling duration = 1500 ms, PLD = 1800 ms, number of C‐L pairs = 20, and acquisition time = 5 minutes 40 seconds. After excitation pulse, a three‐line reference scan was acquired without phase‐encoding blips for phase correction. For CBF quantification, a calibration scan was performed using the same readout but without labeling and background suppression (see Section 2.3.2). For background suppression, a presaturation was applied before the pCASL train, and then two global inversion pulses during PLD, with positions chosen to minimize signal for tissues with T_1_s between 700 and 1400 ms. For 2 participants, high‐resolution ASL scans with double and triple resolution in the partition direction (ie, 2.0 and 1.33 mm) were additionally acquired. The parameters remained the same except for the use of four and six shots, which doubled (40) and tripled (60) the number of C‐L pairs to match the SNR of the lower‐resolution acquisition, resulting in 22‐minute and 48‐minute 52‐second scans, respectively. The need to perform only 2 and 3 times the number repetitions rather than 2^2^ or 3^2^ repetitions is due to the fact that in 3D readouts, increasing the number of acquired k‐space lines also increases SNR. An MPRAGE sequence was acquired with TR/TE/TI = 1700/2.63/900 ms, flip angle = 9°, FOV = 236 × 270 × 194 mm^3^, resolution = 1.05 × 1.05 × 1.1 mm^3^, image matrix = 224 × 256 × 176, and acquisition time = 6 minutes 20 seconds. This study was approved by the research ethics committee of our institution, and written informed consent was obtained from all participants.

### 2.3 Data preprocessing

#### 2.3.1 Motion estimation

To estimate head motion during acquisition, the C‐L image pairs were individually reconstructed in their native resolution and processed with *SPM12* 22 and the *ASL toolbox*.^15^ For this purpose, the *M*_0_ image of each ASL data set was registered to its corresponding T_1_‐weighted (T1w) MR image using *SPM* with default co‐registration parameters. The *ASL toolbox* rigidly registers all control and label images to the calibration scan, while regressing out the potential registration errors caused by the intensity differences of C‐L images.^23^ Finally, the estimated transformations were used for motion correction. As MOCHA relies on perfusion‐weighted data (ie, subtraction of control and label k‐spaces), the motion between control and label data within a pair was neglected, whereas motion between pairs was estimated and corrected. For the other reconstruction methods used for comparison, motion was corrected for each image (both control and label).

#### 2.3.2 Standard image reconstruction and CBF quantification

The standard reconstruction of ASL data was performed using direct inverse Fourier transform. Coil maps were estimated by dividing the MR image from each coil (reconstructed by inverse Fourier transform) by the root sum of squares of all images obtained from all of the coils.^24^ The estimated motion transformations were used to compensate for motion for each control and label image. In cases in which PSF deblurring was applied for the standard reconstructions, a Lucy‐Richardson deconvolution (100 iterations) was performed.^9^ The control and label images were then subtracted and averaged to obtain a perfusion‐weighted image ( $x_{P}$), which was converted into CBF maps (mL/100 g/min) using the following equation^1^:

(5) $$\text{CBF}=\frac{6000\lambda \;\times \;x_{P}\;\times \;\exp\left(-\frac{\mathrm{PLD}}{T_{1,b}}\right)}{2\alpha \;\times \;T_{1,b}\;\times \;x_{M_{0}}\;\times \;\left(1-\exp\left(-\frac{\tau }{T_{1,b}}\right)\right)},$$

where the label duration τ=1500 ms, PLD = 1800 ms, brain‐blood partition coefficient λ=0.9 mL/g, longitudinal relaxation time of blood $T_{1,b}=1650$ ms at 3 T, labeling efficiency α=0.85 as suggested in Alsop et al^1^; and $x_{M_{0}}$ is the *M*_0_ calibration image corrected for TR = 4 seconds. The standard reconstructed images were then corrected for PVE using a 3DLR method implemented in *MATLAB* using a kernel of 5 × 5 × 5 voxels, on the ratio of $x_{P}/x_{M_{0}}$. The FSL FAST tool^25^ was used to estimate high‐resolution GM and WM PV maps from structural images, which were then transformed into the low‐resolution ASL image space using FSL’s *applywarp* with spline interpolation and a super resolution level of 4. In Supporting Information Figure S2, the regions of interest (ROIs) and GM and WM PV maps obtained from the T_1_‐MPRAGE of a participant are shown. The MOCHA method was implemented in *MATLAB* as summarized in Appendix A.

### 2.4 Simulations

A numerical ground‐truth CBF map was simulated by segmenting the T1w MR image (224 × 256 × 176 and 1.05 × 1.05 × 1.1 mm^3^) of subject 1 into WM, GM, and CSF regions using *SPM*. The resulting PV maps were then used to generate a CBF map by multiplying the tissue blood flows of 65 and 20 mL/100 g/min by the GM PV and WM PV maps, respectively.^26^ Furthermore, a 1.34‐mL circular WM hyperperfused lesion with a blood flow of 100 mL/100 g/min, a regional hyperperfusion (78.9 ± 8.6 mL/100 g/min), and hypoperfusion (36.6 ± 3.8 mL/100 g/min) were created to evaluate the effect of mismatches between anatomical and perfusion information on the reconstructed CBF maps. To simulate realistic high‐resolution control, label and *M*_0_ images, the *M*_0_ and the first control k‐space images of subject 1 were reconstructed in the resolution space of the T1w image using the method described in Equation 4.

**Figure 2:**
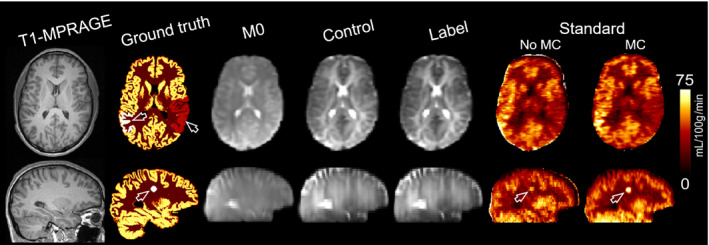
Simulated brain arterial spin labeling phantom comparing the ground‐truth CBF map with the low‐resolution cerebral blood flow (CBF) maps reconstructed using the standard method with motion correction (MC) and without motion correction (No MC)

Using the simulated high‐resolution CBF, *M*_0_ and control images, a high‐resolution label image was then created based on Equation 5 with the default parameters. The control, label, and *M*_0_ images were then resampled into the resolution of ASL data (the same as our in vivo data). In these simulations, 20 pairs of C‐L images were considered. To simulate motion, each image was incrementally rotated, leading a maximum angular drift of 3° between the first and last C‐L pair and translation of 15 mm (see Supporting Information Figure S1). The images were then downsampled to match the native resolution of our in vivo ASL data, blurred in the partition direction using a 6‐mm FWHM Lorentzian filter, modulated by the calculated coil sensitivity maps, and Fourier‐transformed to obtain a multichannel k‐space data set. Gaussian noise was added to the k‐space data to obtain an SNR of 15 dB. Finally, the motion transformation of each C‐L pair was estimated with the procedure described in Section 2.3.1. Figure 2 shows the high‐resolution T1w and CBF images together with the simulated low‐resolution *M*_0_, first control and label images, and the CBF maps estimated by the standard method with and without motion correction.

### 2.5 Evaluation and parameter selection

The standard, 3DLR, and MOCHA methods were evaluated for quantification of CBF in WM, cortical GM, and different subcortical GM regions of the simulated and in vivo data sets. The T_1_‐MPRAGE images were parcellated into GM, WM, thalamus, caudate, putamen, pallidum, and hippocampus using *FreeSurfer*.^27^ For simulations, the reconstruction methods were evaluated based on the mean CBF in different parcellated regions. For 3DLR, depending on the ROI, the most appropriate of either the GM or WM PV–corrected maps was used to extract the mean values. The normalized RMS error (NRMSE) was defined as

(6) $$NRMSE_{i}\left(\%\right)=100\;\times \;\sqrt{\frac{\sum_{j\in ROI_{i}}{\left(x_{j}-x_{j}^{\mathrm{GT}}\right)}^{2}}{\sum_{j\in ROI_{i}}{\left(x_{j}^{\mathrm{GT}}\right)}^{2}}},$$

where $x_{}^{\mathrm{GT}}$ is the ground‐truth CBF map. For the simulated data, the β parameter of the MOCHA was optimized based on minimization of NRMSE over the whole brain, whereas the rest of the parameters were empirically set to σ=0.15, N= 3 × 3 × 3, and $N_{\mathrm{it}}=100$ iterations of the steepest decent algorithm. The same parameters and the same β were then used for in vivo reconstructions. For simulations, the PSF through‐plane FWHM was set to 1.5 times the slice thickness to mimic an acquisition with T_2_ decay during the 3D‐GRASE echo train. For in vivo data, the PSF was modeled as a Lorentzian with FWHM of one slice thickness. The PSF estimation was performed using autocorrelation of the residuals as described in Chappell et al.^28^ In this method, multiple C‐L differences are mean‐subtracted to generate voxel‐level residuals. A one‐dimensional series of residuals in the superior–inferior direction were then obtained by averaging across measurements, as well as in the anterior–posterior and left–right directions. The autocorrelation of these residuals was fitted with the autocorrelation of a Lorentzian, giving an estimate of the PSF width.

To evaluate the performance of the MOCHA method for accelerated ASL imaging, an in vivo data set was reconstructed with a retrospectively reduced number of 10 and 5 C‐L pairs.

All results were evaluated in T1w space.

## 3 RESULTS

### 3.1 Simulations

**Figure 3:**
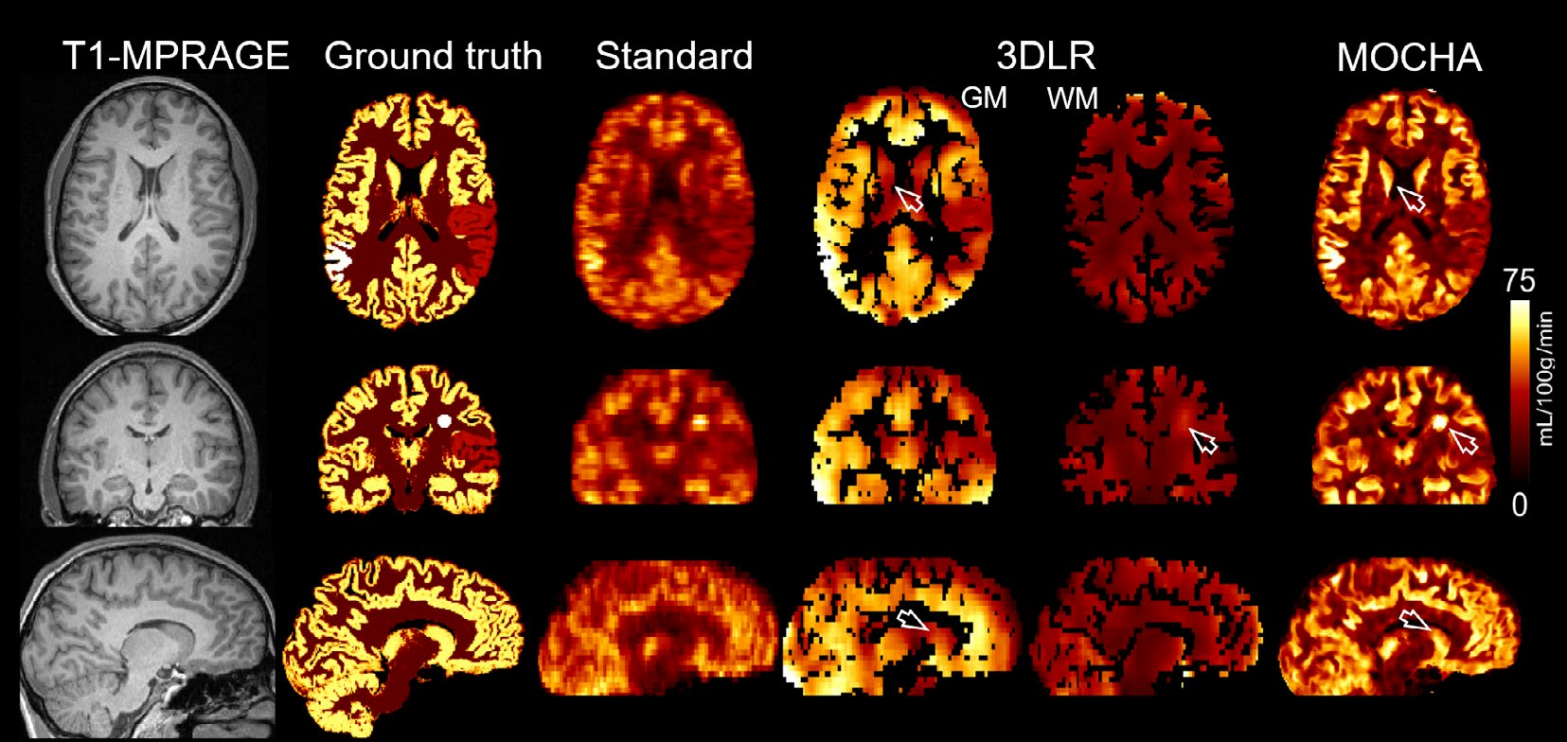
Results for the reconstruction of simulated data for a motion‐corrected CBF map obtained from the standard method, corrected for partial‐volume averaging of gray matter (GM) and white matter (WM) using the 3D linear regression (3DLR) method and reconstructed using the MOCHA high‐resolution method. The arrows point to where MOCHA outperforms 3DLR in the caudate and simulated WM lesion. On the low‐resolution 3DLR data, the boundaries of the simulated GM lesions are also not well defined

Figure 3 shows the simulation results of the standard, 3DLR, and MOCHA reconstruction methods (all including motion correction). As shown, the standard method notably suffers from PVE and loss of details. The 3DLR method separates the GM and WM CBFs for each voxel of the standard CBF map, resulting in partial recovery of estimated CBF in the GM, although at the cost of loss of boundaries in the simulated hyper/hypo perfused regions, severe smoothing and suppression of the simulated lesion (see arrows). It was also apparent that some deep GM structures such as putamen and caudate were not appropriately PV‐corrected by the 3DLR method. In contrast, MOCHA showed tissue boundaries and recovered deep GM CBF to a good extent. Due to the severe blurring introduced and low acquisition resolution simulated, uniform intensity of GM CBF across uniform regions (such as the thin cortical ribbon) could not be achieved. Importantly, the hyperperfused lesion and hyperperfused/hypoperfused regions were well delineated, despite there being no corresponding structure on the anatomical image used for guidance.

Supporting Information Figure S3 shows the results of a similar analysis as in Figure 3 without the motion‐correction step, showing substantial degradation of the reconstructed maps. Importantly, no motion artifacts are apparent for MOCHA in Figure 3 with the relatively large simulated motion, despite its neglect of within‐pair motion.

**Figure 4:**
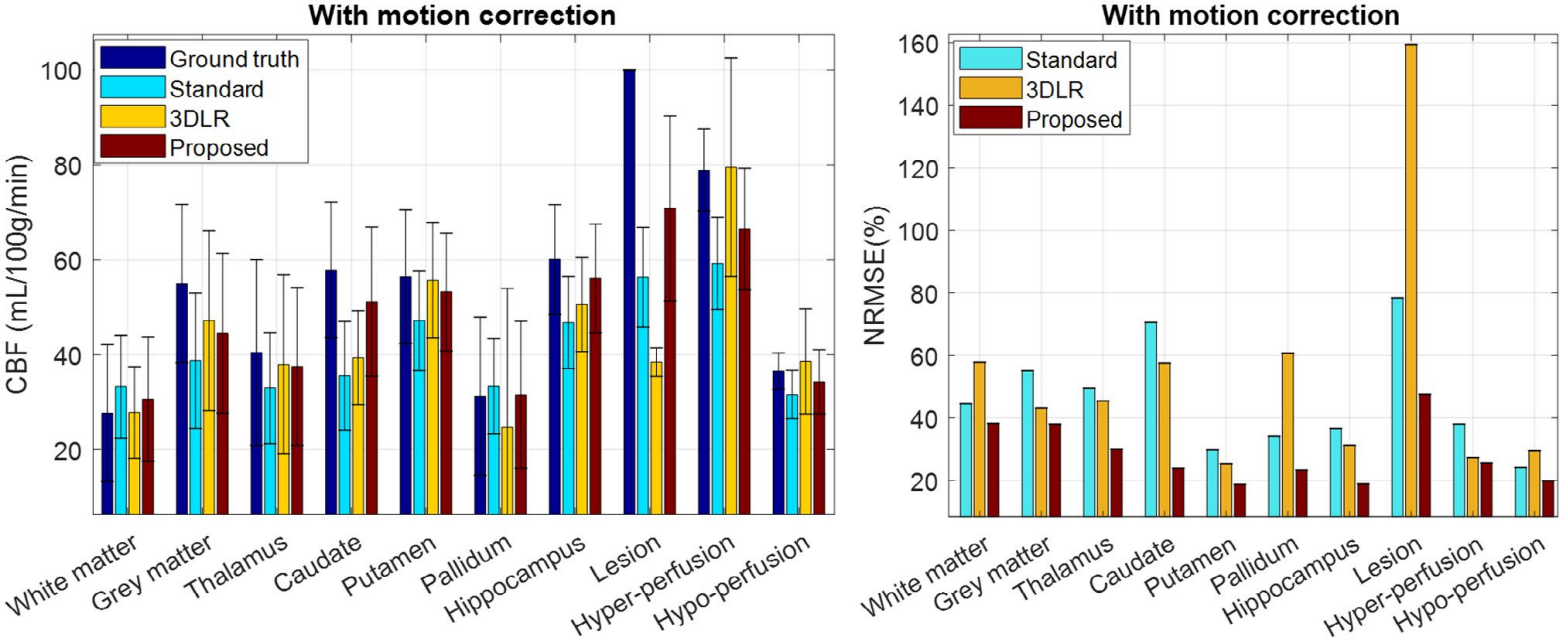
The mean and SD of CBF values estimated by the studied methods in different regions of the simulated brain phantom with motion correction. Note the GM region only contains cortical GM. Abbreviation: NRMSE, normalized RMS error

Figure 4 shows the performance of the methods in terms of mean and SD of CBF values and NRMSEs in different regions of the brain. The corresponding values (with and without motion‐correction step) are summarized in Supporting Information Tables S1 and S2.

In terms of mean ROI values, the standard reconstruction overestimates CBF in WM and pallidum, and underestimates CBF in all others with −44%, −25%, and −14% in the WM lesion and GM hyper/hypo regions, respectively (ROIs of mismatch between anatomy and perfusion). The MOCHA technique is closer to the ground‐truth values than the standard reconstruction in all cases (with −29%, −16%, and −6% in the mismatch ROIs). Three‐dimensional LR is closer to the true values than the standard method in all regions except the pallidum and WM lesion (with −62%, +1%, and +5% in the mismatch ROIs). The slightly better match to the ground truth of the 3DLR compared with MOCHA for cortical GM (−14% vs. −19%) and WM (+1% vs. +10%) is due to the fact that the 3DLR values reported here explicitly contain only contributions from either GM or WM 3DLR maps. The MOCHA technique shows more accurate CBF values than 3DLR for caudate and pallidum (20% improvements), hippocampus (9% improvement), and particularly in the GM hyperperfusion. Three‐dimensional LR is slightly better than MOCHA in the putamen (−1 vs. −6%) and in the GM hyperperfusion mismatch region. Despite 3DLR showing mean ROI values closer to the ground truth in some regions, MOCHA provided lower voxel‐level NRMSEs in all regions (with reductions of 112%, 2%, and 10% in the mismatch ROIs vs. 3DLR). The NRMSE for 3DLR was higher than for the standard reconstruction in the WM, WM lesion, and GM hypoperfusion regions. Removing the motion‐correction step causes an increased NRMSE for all methods/regions, and a general CBF underestimation, particularly in all the anatomical/perfusion mismatch regions.

Supporting Information Figure S4 presents similar reconstructions as in Figure 3, but with additional PSF deconvolution for the standard and 3DLR methods using the same PSF used for MOCHA. The images show improved contrast for the standard reconstruction method, at the expense of noise amplification. Supporting Information Figure S5 shows that PSF deblurring slightly changed the NRMSE of standard reconstruction (on average by 4.7% reduction, variable across ROIs); for the 3DLR method there were small reductions in WM lesion, cortical GM NRMSE and deep GM NRMSE, with a slight increase in the GM mismatch regions and WM NRMSE.

Supporting Information Figure S6 shows CBF profiles for the studied methods with respect to ground truth. As shown, PSF deblurring amplified the noise for the standard reconstruction, and slightly increased the CBF for 3DLR in GM hyperperfusion. MOCHA, which takes PSF into account in the reconstruction, followed the true profiles more closely.

Supporting Information Figure S7 shows the NRMSE performance of the MOCHA as a function of the regularization parameter β for different regions of the simulated brain phantom. In Supporting Information Figure S8, the MOCHA reconstructions for different β values are shown. Supporting Information Table S3 summarizes the results and highlights the β values that result in the lowest NRMSE in each region. The results show that as the β increases, the errors in the GM and especially WM reduce, although at the expense of increasing errors in the WM lesion. The value of β=20 was chosen for minimal errors in whole brain, a good compromise in the simulated anatomical/perfusion mismatch regions. The same value was used for the in vivo data.

The performance of the 3DLR method was also evaluated as a function of kernel size. As shown in Supporting Information Figure S9, by increasing the kernel size the GM CBF maps are smoother and have fewer details. However, the quantitative results show that mean WM reduces very slightly for larger kernel sizes, while the GM CBF is stable. Hippocampal and deep GM CBF values tend to increase very slightly (ie, better overall PVC performance in these regions). At the same time, the WM lesion’s CBF notably reduces. Hence, as mentioned earlier, in this study a kernel of 5 × 5 × 5 voxels was used, which provided a balanced performance for the 3DLR method for the WM lesion and small GM structures.

### 3.2 In vivo data

**Figure 5:**
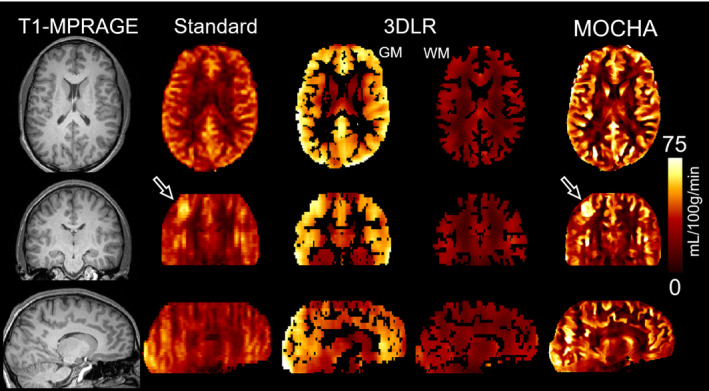
Anatomical image and CBF results for subject 1 calculated using the standard, 3DLR, and MOCHA reconstruction methods. Note that data from this subject were also used for simulations. The arrows indicate an area of apparent local high perfusion

**Figure 6:**
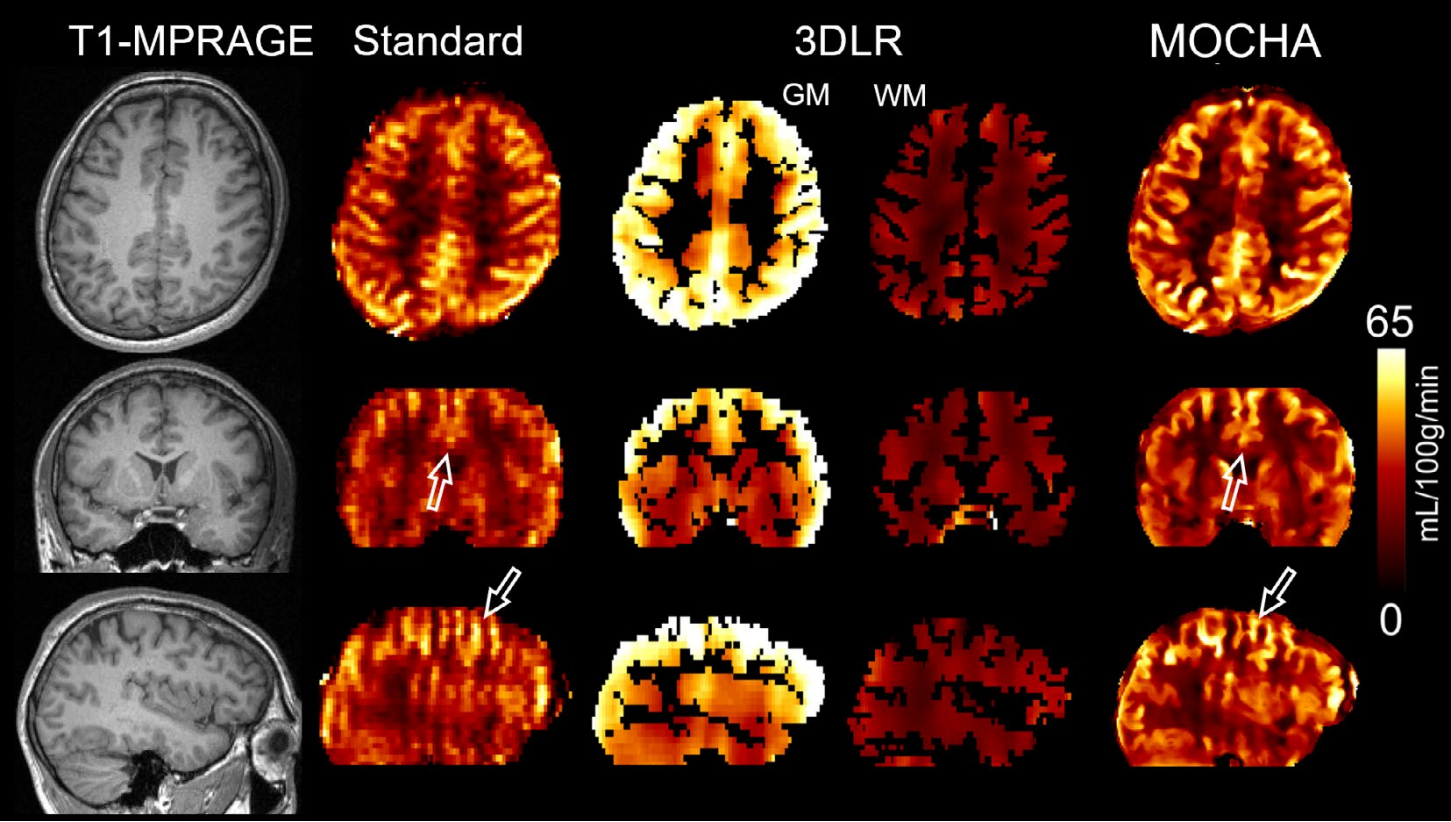
Anatomical image and CBF results for subject 2 calculated using the standard, 3DLR, and MOCHA reconstruction methods

**Figure 7:**
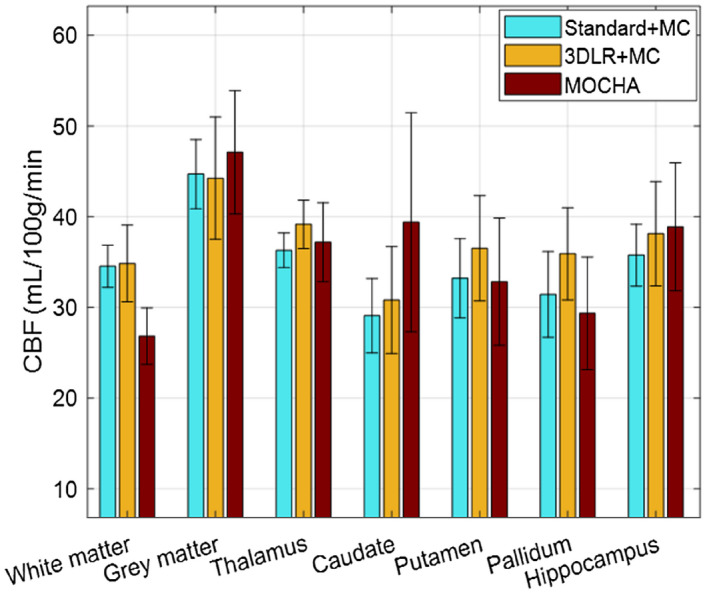
Cerebral blood flow results averaged over four in vivo data sets for standard, 3DLR, and MOCHA reconstruction methods. The error bars show the SD of the mean CBF values calculated for each subject in each region

Figures 5 and 6 show the results of 2 subjects, comparing different methods. Supporting Information Figures S10 and S11 show similar results for another 2 subjects. All of the in vivo data were motion‐corrected. These results show that the standard CBF maps suffer PVE, especially in the partition‐encoding direction. The 3DLR method results in increased GM CBF values, although at the expense of some loss of details, including smoothing of the apparent local high perfusion indicated by arrows in Figure 5. In comparison, the MOCHA method appears to correct for PVE while preserving local hyperperfusions and recovering details in the partition direction (see coronal and sagittal views). Figure 7 shows the quantitative performance of the reconstruction methods in different regions of the brain, averaged over all 4 subjects (values found in Supporting Information Table S4). Similarly to the simulation results, in these in vivo data, MOCHA reduces the WM CBF and increases CBF in most GM regions.

**Figure 8:**
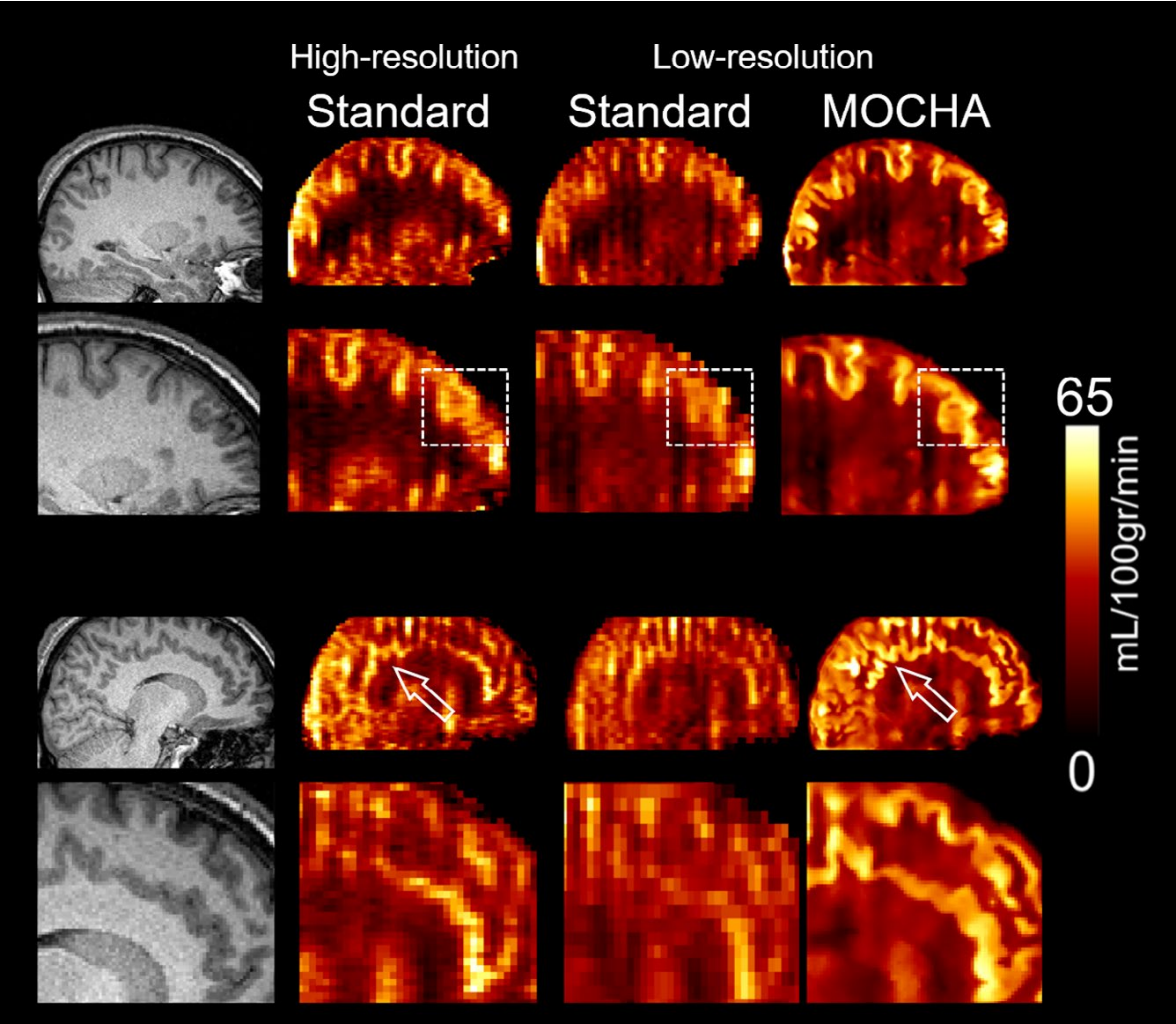
Anatomical image and CBF maps from standard‐resolution (4 × 4 × 4 mm^3^; 5‐minute 40‐second acquisition; standard and MOCHA reconstructions; right) and doubled resolution in the inferior–superior direction (high resolution; 4 × 4 × 2 mm^3^; 22‐minute acquisition; standard reconstruction; left) for subject 5. Note that due to the sequential nature of the acquisitions, there might be physiological differences between low‐resolution and high‐resolution CBF maps

**Figure 9:**
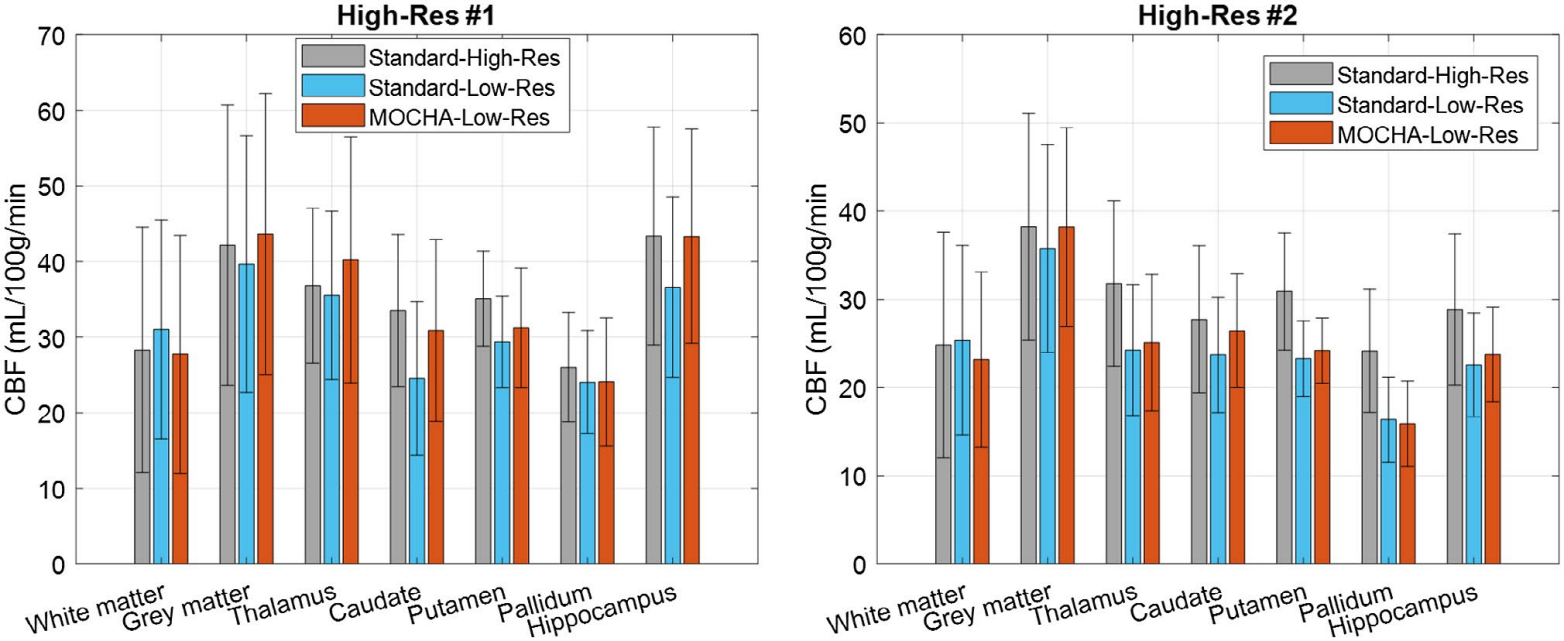
Region of interest–averaged CBF results for the double‐resolution (left) and triple‐resolution (right) arterial spin labeling scans. The error bars show the standard deviations over each region

Figure 8 and Supporting Information Figure S12 show the MOCHA reconstruction of the standard low‐resolution data of subjects 5 and 6 compared with their corresponding higher‐resolution data. As shown previously, MOCHA enhances the anatomical tissue boundaries. Most importantly, many details of the high‐resolution data that are lost in the standard low‐resolution reconstruction have been reliably recovered in the MOCHA reconstruction. Quantitative analysis of these results for the 2 volunteers are shown individually in Figure 9, and the values averaged over the 2 volunteers are found in Supporting Information Table S5. The MOCHA method not only enhances the visual appearance of the low‐resolution CBF maps but also improves their quantitative accuracy toward the values found in the reference high‐resolution CBF map for most ROIs. The averaged cortical GM CBF values for these volunteers were 40.2, 37.7, and 40.7 mL/100 g/min for the high‐resolution standard, low‐resolution standard, and low‐resolution MOCHA reconstructions, respectively.

**Figure 10:**
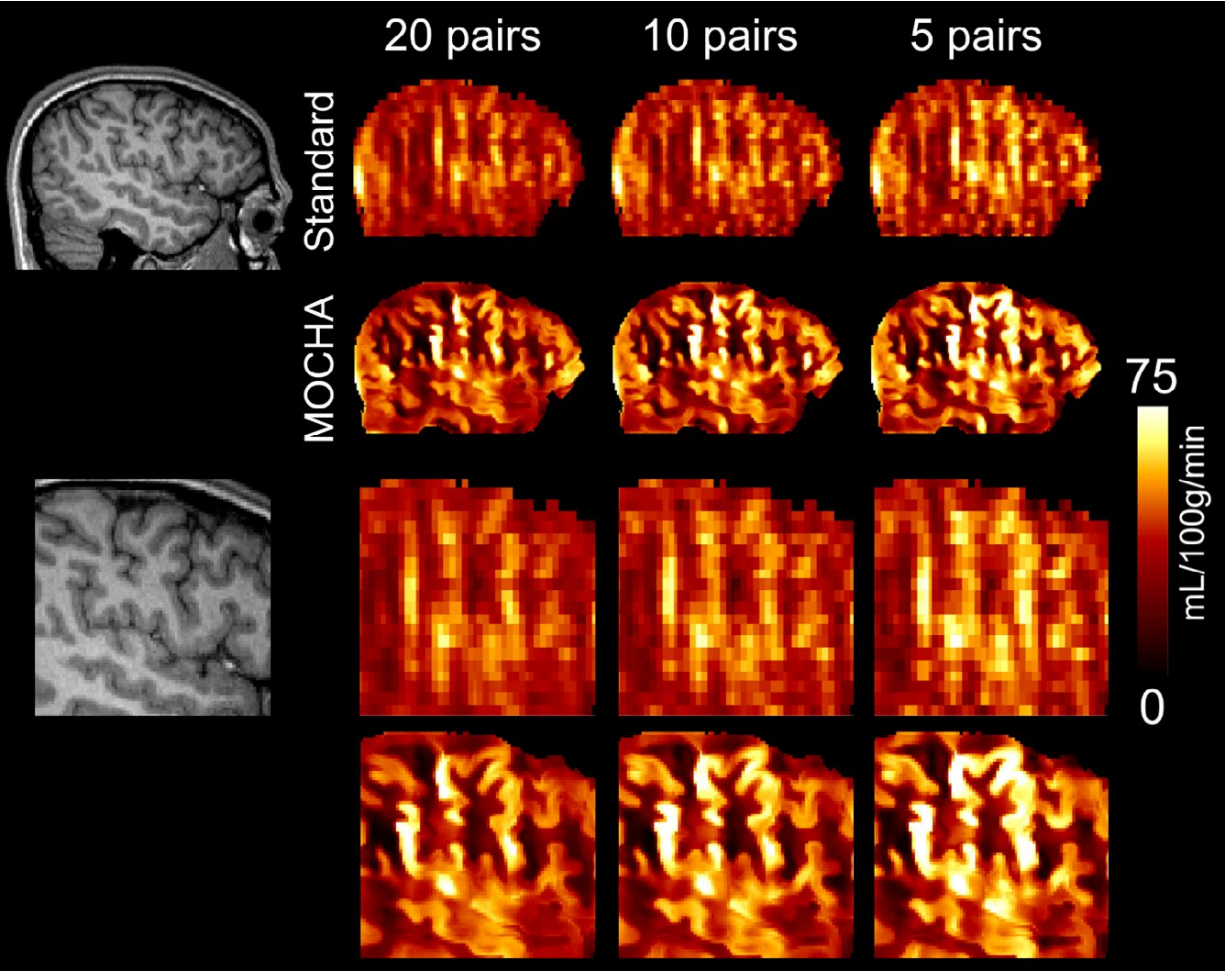
Cerebral blood flow results for subject 4 calculated using standard and MOCHA reconstruction methods using different numbers of control‐label pairs (ie, 1‐20, 1‐10, and 1‐5)

The performance of MOCHA was further evaluated for acquisitions with a lower number of C‐L pairs (or repeats), which would entail reduced scan time and correspondingly reduced SNR. For this purpose, a data set was retrospectively reduced to 10 and 5 C‐L pairs out of 20 pairs, equivalent to SNR reductions of 1.4 and 2, respectively. Figure 10 compares the reconstruction results of the standard and MOCHA methods. As shown, for a lower number of C‐L pairs, the standard CBF map appears slightly noisier compared with the reference 20‐pairs image, whereas MOCHA shows more consistent maps. Supporting Information Table S6 summarizes the quantitative performance of the methods. The results show a slight GM CBF decrease with the increase of C‐L pairs used, which could be potentially attributed to a physiological decrease of CBF during the 6‐minute acquisition. The expected acquisition times for 5‐pair and 10‐pair acquisitions are, including dummy scans and M_0_ data collection, 100 seconds and 180 seconds compared with 340 seconds of the reference 20‐pair scan.

The performance of MOCHA was also evaluated for undersampled ASL scans. For this purpose, the k‐space data of subject 4 were retrospectively undersampled at two levels (acceleration factor R of 2 and 4) in the phase‐encoding (anterior–posterior) direction. The performance of MOCHA was then compared with the standard CBF maps reconstructed using SENSE and SENSE with total variation regularization.^29^ As shown in Supporting Information Figure S13, MOCHA reduces noise and undersampling artifacts and maintains an image quality similar to fully sampled data, demonstrating good potential for undersampled acquisitions with highly accelerated acquisition times.

## 4 DISCUSSION

In this study, the proposed MOCHA reconstruction framework was compared with the 3DLR PVC method. The 3DLR method separates the GM and WM signals within each voxel of the standard low‐resolution CBF maps by solving a system of equations in which the GM and WM PV fractions are known coefficient values. This method assumes that all voxels in the neighborhood (kernel) of a given voxel have the same GM and WM CBF values; hence, the system is uniquely solved by a least‐squares estimator, although at the expense of smoothing image details, as shown here and by others. Recently, a Bayesian approach was proposed to solve the underdetermined system by using the kinetic model of the GM‐WM signals in multi‐PLD ASL acquisitions together with a previous modeling of the spatial correlation of kinetic parameters.^6^ Because, in our study, the data were acquired with single PLD and perfusion was quantified using Equation 5, as recommended by Alsop et al,^1^ rather than using a kinetic curve fitting as in Chappell et al,^6^ the Bayesian method was not included to avoid any discrepancy caused by the perfusion estimation method. However, Oliver et al compared the 3DLR (3 × 3 × 3 kernel) and Bayesian methods in 6 healthy controls.^17^ Despite comparable mean GM‐CBF values, the Bayesian method retained structural details at the expense of increased sensitivity to noise. Recently, Zhao et al compared a 2DLR (3 × 3 kernel) with the Bayesian method using comprehensive simulations and in vivo data,^30^ and similar results were observed.

In contrast to these two methods, MOCHA aims to reconstruct directly a high‐resolution CBF map corrected for different sources of PVE, such as large voxel sizes, PSF, and motion blurring. The MOCHA technique uses all C‐L pairs to reconstruct a single perfusion‐weighted image, such that the averaging is performed during reconstruction rather than after reconstruction of the individual C‐L pairs. The same idea was recently used by Spann et al^31^ to explore temporal redundancy and spatial similarity of the C‐L pairs for ASL reconstruction. As expected, in simulations both 3DLR and MOCHA produced averaged GM/WM CBF values closer to ground truth than the standard reconstruction. The MOCHA method provided sharper anatomical boundaries, whereas 3DLR showed increased blurring. The 3DLR mean values were slightly closer to the ground truth in cortical GM and hyperperfused GM, whereas MOCHA performed a lot better in the WM hyperperfusion region. Furthermore, MOCHA provided the lowest NRMSE in all of the analyzed regions.

The MOCHA technique relies on anatomical images for regularization of high‐resolution CBF maps. Although this improves the quality of the reconstructed CBF maps, we are aware that some functional features (ie, geometry of flow territories, vascular artifacts) influencing CBF are not captured by anatomy (ie, GM/WM/CSF PV fractions or tissue appearance on T1w images); hence, any method (including LR) relying on anatomical information for PVE correction could lead to partly biased results, and a high‐resolution perfusion signal cannot be completely recovered solely by using anatomical information. At the same time, we have shown that by combining low‐resolution perfusion data and high‐resolution anatomical information, MOCHA does go some way toward correcting functional maps for PVE and blurring, and thus improving their spatial and quantitative accuracy. We have considered a number of scenarios to demonstrate this by simulating anatomy/perfusion mismatches, (ie, hyperperfusion/hypoperfusion in GM and WM regions with no corresponding structural abnormalities). Although MOCHA’s quantitative accuracy varies depending on the region, it always offers an improvement compared with the standard reconstruction, and in all cases provides an improved preservation of boundaries and the lowest voxel‐level errors (NRMSE) compared with the ground truth. In these situations, 3DLR’s quantitative performance is also variable, and not being able to rely on PV information, it is inherently affected by large blurring, the extent of which depends on the chosen kernel size. As an additional comparison, we also show in Supporting Information Figure S14 a CBF map obtained from the combination of the 3DLR GM and WM maps weighted by GM and WM PVFs.

We have also used in vivo data sets acquired with high‐resolution protocols as high‐quality references to validate the MOCHA reconstructions obtained from standard low‐resolution (4 × 4 × 4 mm) 6‐minute acquisition time data sets. To obtain these references, we doubled and tripled the slice resolution, requiring long acquisitions of 22 minutes and 49 minutes, respectively, for full k‐space sampling with equivalent SNR. It was apparent that many of the details in the long‐acquisitions/high‐resolution CBF maps are in fact well reproduced in the MOCHA images reconstructed from low‐resolution data. This suggests an effective resolution improvement, which is beyond purely visual improvement.

Finally, we have provided evidence that MOCHA‐reconstructed CBF maps are robust to a severe reduction in the number of C‐L pairs (averages) collected, with reductions tested up to a factor of 4, and k‐space undersampling, also tested up to a speed‐up factor of 4. Although there are clear advantages, MOCHA nonetheless has some limitations. Inclusion of PSF and downsampling and using an anatomical prior leads to only partial recovery of the lost high‐frequency information. In Boussion et al,^32^ a similar idea of transferring the high‐frequency information from structural MR to low‐resolution emission tomography data has been proposed without segmentation of the MR image. The PSF was assumed to be shift‐invariant and motion‐independent. Following Elad and Hel‐Or,^33^ the blurring ***B*** was therefore used as the front‐end operator in Equation (1), which allows the motion transformation and downsampling operators to be merged into one single spatial transformation, reducing the computational burden of the model.

As tissue boundaries of the anatomical data influence the MOCHA reconstruction of the perfusion images, any motion left unaccounted for, as well as any distortions or misregistrations affecting the alignment of the structural and perfusion data, can all negatively affect the accuracy of the reconstruction. Although a number of steps were taken to minimize these effects, further improvements are possible and will be undertaken. The current MOCHA implementation only takes into account motion occurring between C‐L pairs and neglects motion during each acquisition/pair. For the continuous motion in our simulations and our healthy volunteers, the current interframe motion correction appeared to be sufficient, but more complex motion patterns may occur, especially for non‐cooperative patients. A possible solution to address frame‐by‐frame motion is to reconstruct a motion‐corrected control image from all control data and likewise for all label data, and then to perform a postreconstruction subtraction. Additionally, it is possible to identify motion‐corrupted “outlier” pairs (such as by using ENABLE^34^) and remove them from the analysis. In our data with limited PSF blurring, the *M*_0_ to T1w rigid‐body registration produced satisfactory registration. However, we are aware that, in general, for 3D‐GRASE ASL, which suffers from T_2_ blurring, registering CBF maps with GM PV maps, as in Mutsaerts et al,^35^ has been shown to be more reliable; this method is therefore recommended for a more general application of MOCHA.

In this paper, susceptibility‐induced geometric distortions were not included in our forward model. Instead, they were minimized by the 2‐fold segmentation in the phase‐encoding (anterior–posterior) direction. However, small, residual, localized spatial mismatches between anatomical and perfusion images can remain. Future work should include estimation and correction of the spatial distortions, such as using reversed gradient (blip up/down) acquisitions, further reducing this potential source of error.

All of these various misalignment errors discussed can cause some local or global CBF errors. However, their magnitude also depends on the strength of the regularization ( β) and the shape of the Gaussian similarity coefficients ( σ). In this work, σ,N(neighborhood size) and the number of iterations were chosen heuristically. Larger values of σ reduce the impact of MR information, as the resulting weights will tend to be more uniform. We have found σ values in the range of 0.1‐0.3 result in appropriate weighting of the structural information from T1w images. The value of N was set to 3 × 3 × 3, as in our CPU‐based implementation. Larger neighborhoods are memory demanding, and based on our previous experience, larger neighborhoods do not notably lead to improved regularization. We used a large number of iterations to ensure that the steepest descent algorithm (which improves upon gradient descent by step‐size optimization) converges to at least a fixed point. In our experience, the most important hyperparameter is β, which was optimized.

For the main results, we compared MOCHA to 3DLR with a 5 × 5 × 5 voxel kernel and no PSF modeling. The kernel size of the LR method affects its performance in terms of robustness to noise,^30^ geometric distortion,^36^ and of repeatability between scans.^37^ This was chosen based on our tests with kernels ranging from 3 × 3 × 3 to 9 × 9 × 9 voxels. Taking into account the PSF for 3DLR only provided a small reduction in NRMSE in GM ROIs and the WM lesion, but not in the GM hypoperfusion/hyperperfusion regions; therefore, for the main results, PSF modeling was not included, which is consistent with the most common use of 3DLR in the existing literature. However, we also acknowledge that the PSF should always be taken into account when downsampling^38^ and preparing the data for 3DLR.^36^ One way to estimate PSF is through simulation of the vascular signal through the pulse sequence, as in Vidorreta et al.^40^

Admittedly, the 3DLR performance in some deep GM regions could have been improved with a different PV estimation method, such as the recently developed tissue probability maps with better subcortical performance.^39^ However, this would not have improved 3DLR results in areas of anatomical/functional mismatches and highlights the dependence of the 3DLR on PV tissue fraction and its estimation method.

We have not at this stage examined the noise properties of the final CBF images obtained, and finding the endpoint noise properties would be an interesting topic for future work. This would require consideration of the noise propagation from the M0 and C‐L images to the final reconstructed MOCHA image.

Our method is computationally intensive due to the inclusion of motion, spatial mapping, and PSF operators in the forward model. The computation time for 1 and for 100 iterations in *MATLAB* R2017a (running on a 20‐core Intel Xeon 3.10‐GHz workstation, for a data set of 20 averages) was approximately 1 minute and 1.5 hours, respectively. The objective function of MOCHA is convex and continuously differentiable; hence, the steepest descent algorithm guarantees convergence to the global minimizer, regardless of the initial estimate.

To our knowledge, MOCHA is the first fully model‐based high‐resolution reconstruction method for ASL data. Our results show a good performance of MOCHA in simulations including areas of anatomical/perfusion mismatch. In vivo data demonstrated that MOCHA can reliably reconstruct high‐resolution CBF maps from standard low‐resolution data sets, featuring many of the details observed in the higher‐resolution data sets (which require impractically long acquisition times). The robustness of the reconstruction to short acquisitions and/or undersampling was also demonstrated. The actual clinical benefits of MOCHA will be evaluated in collaboration with radiologists, using the patient data presented with and without MOCHA reconstruction.

## 5 CONCLUSIONS

Simulation and in vivo data results demonstrate that the proposed direct high‐resolution CBF map reconstruction method effectively corrects motion and PVE. The MOCHA technique is advantageous in preservation of structural details and hypoperfused/hyperperfused regions. The MOCHA framework has the potential to improve the diagnostic confidence and applicability of current ASL protocols in clinical practice.

## Supporting information


**FIGURE S1** Simulated motion (translation and rotation) in our simulation data set
**FIGURE S2** Regions of interest (ROIs) and GM partial‐volume (PV) estimates obtained from the parcellation of the T_1_‐MPRAGE MR image using the *Freesurfer* and FSL (*FAST* and *applywarp*) software. The arrow points to the pallidum that has been erroneously identified as WM
**FIGURE S3** Same as Figure 3, except for the omission of the motion‐correction step
**FIGURE S4** Same as Figure 3, except for the addition of point‐spread‐function deblurring for the standard and 3DLR methods. Note: The motion‐correction step is included for all methods
**FIGURE S5** Effect of point‐spread‐function (PSF) deblurring on NRMSE performance of the standard and 3DLR methods for simulations
**FIGURE S6** Cerebral blood flow profiles of the studied reconstruction methods through simulated WM lesion and GM hyperperfusion
**FIGURE S7** The NRMSE performance of MOCHA in different regions of the simulated brain phantom as a function of the regularization parameter β
**FIGURE S8** Reconstruction results of the MOCHA method as a function of regularization parameter
**FIGURE S9** Effect of kernel size on the qualitative (top) and quantitative (bottom) performance of the 3DLR method in comparison with the standard method and the ground‐truth simulated brain phantom
**FIGURE S10** Cerebral blood flow results for subject 3 calculated using the standard, 3DLR, and MOCHA reconstruction methods. The arrows point to the most notable differences between MOCHA and standard reconstruction methods
**FIGURE S11** Cerebral blood flow results for = subject 5 calculated using the standard, 3DLR, and MOCHA reconstruction methods. The arrows point to some regions where there are notable differences between MOCHA and standard reconstruction methods
**FIGURE S12** Anatomical image and CBF maps from standard‐resolution acquisition (4 × 4 × 4 mm^3^; 5‐minute 40‐second acquisition), standard, and MOCHA reconstructions; right) and tripled resolution in the slice direction (high resolution, 4 × 4 × 1.33 mm^3^; 49‐minute acquisition; standard reconstruction only; left) data sets for subject 6
**FIGURE S13** Cerebral blood flow results of subject 4 calculated using the standard and MOCHA reconstruction methods for different undersampling factors (R). The arrow shows an undersampling artifact. Standard and SENSE reconstructions show increased noise as R increases. Total‐variation SENSE (TV‐SENSE) also shows visible changes between R = 2 and R = 4. The MOCHA method shows the highest visual consistency between reconstructions at R = 1, R = 2, and R = 4
**FIGURE S14** Top: Similar to Figure 3, including GM and WM partial‐volume fractions (pGM and pWM) and a combined image corresponding to pGM* 3DLR‐CBF_GM_ + pWM* 3DLR‐CBF_WM_. As shown in the combined image, the cortex appears thinned and the contrast of the synthetic WM lesion is substantially decreased. Bottom: Quantitative comparison of the method; as shown, MOCHA achieves better performance in deep GM structures, and the combined method results in CBF values very similar to the standard method
**TABLE S1** Quantitative performance of the standard, 3DLR, and MOCHA methods in terms of CBF (mean ± SD) in different regions of the simulated brain phantom with and without motion correction
**TABLE S2** The NRMSE (%) of the studied methods with and without motion correction in different regions of the simulated brain phantom
**TABLE S3** The NRMSE performance of MOCHA in different regions of the simulated brain phantom as a function the regularization parameter β
**TABLE S4** Mean and steepest decent of CBF values averaged over 4 healthy subjects
**TABLE S5** Mean and steepest decent of CBF values averaged over the 2 high‐resolution healthy subjects
**TABLE S6** Mean and steepest decent values of CBF maps for a subject calculated for different control‐label pairs
