# Supplementary material for: Motion‐corrected and high‐resolution anatomically assisted (MOCHA) reconstruction of arterial spin labeling MRI
Source: Magn Reson Med. 2020 Mar 3;84(3):1306–20. doi: 10.1002/mrm.28205 (PMC8614125; doi:10.1002/mrm.28205)
Supplement: Supplementary file 1 — FIGURE S1 Simulated motion (translation and rotation) in our simulation data set FIGURE S2 Regions of interest (ROIs) and GM partial‐volume (PV) estimates obtained from the parcellation of the T1‐MPRAGE MR image using the Freesurfer and FSL (FAST and applywarp) software. The arrow points to the pallidum that has been erroneously identified as WM FIGURE S3 Same as Figure 3, except for the omission of the motion‐correction step FIGURE S4 Same as Figure 3, except for the addition of point‐spread‐function deblurring for the standard and 3DLR methods. Note: The motion‐correction step is included for all methods FIGURE S5 Effect of point‐spread‐function (PSF) deblurring on NRMSE performance of the standard and 3DLR methods for simulations FIGURE S6 Cerebral blood flow profiles of the studied reconstruction methods through simulated WM lesion and GM hyperperfusion FIGURE S7 The NRMSE performance of MOCHA in different regions of the simulated brain phantom as a function of the regularization parameter β FIGURE S8 Reconstruction results of the MOCHA method as a function of regularization parameter FIGURE S9 Effect of kernel size on the qualitative (top) and quantitative (bottom) performance of the 3DLR method in comparison with the standard method and the ground‐truth simulated brain phantom FIGURE S10 Cerebral blood flow results for subject 3 calculated using the standard, 3DLR, and MOCHA reconstruction methods. The arrows point to the most notable differences between MOCHA and standard reconstruction methods FIGURE S11 Cerebral blood flow results for = subject 5 calculated using the standard, 3DLR, and MOCHA reconstruction methods. The arrows point to some regions where there are notable differences between MOCHA and standard reconstruction methods FIGURE S12 Anatomical image and CBF maps from standard‐resolution acquisition (4 × 4 × 4 mm3; 5‐minute 40‐second acquisition), standard, and MOCHA reconstructions; right) and tripled resolution in the slice direction (high resol [file MRM-84-1306-s001.docx]

**Supporting Information**

**Tables**

**Table S1.** Quantitative performance of the standard, 3DLR and MOCHA methods in terms of cerebral blood flow (mean ± standard deviation) in different regions of the simulated brain phantom with and without motion correction.

| ROI |  |  | No motion correction | | |  | With motion correction | | |
| --- | --- | --- | --- | --- | --- | --- | --- | --- | --- |
|  | Ground truth |  | Standard | 3DLR | MOCHA |  | Standard | 3DLR | MOCHA |
| White matter | 27.7 ± 14.5 |  | 33.9 ± 11.9 | 29.9 ± 10.8 | 34.4 ± 14.2 |  | 33.3 ± 10.9 | 27.9 ± 9.6 | 30.7 ± 13.0 |
| Grey matter | 55.0 ± 16.7 |  | 36.6 ± 16.5 | 42.9 ± 20.2 | 39.0 ± 17.1 |  | 38.8 ± 14.2 | 47.2 ± 19.0 | 44.6 ± 16.8 |
| Thalamus | 40.5 ± 19.6 |  | 32.0 ± 10.8 | 34.5 ± 18.1 | 34.2 ± 14.5 |  | 33.0 ± 11.7 | 38.0 ± 18.8 | 37.6 ± 16.6 |
| Caudate | 57.9 ± 14.3 |  | 31.9 ± 11.1 | 33.9 ± 10.8 | 39.7 ± 13.2 |  | 35.6 ± 11.4 | 39.4 ± 9.9 | 51.2 ±15.7 |
| Putamen | 56.5 ± 14.1 |  | 49.4 ± 9.6 | 53.1 ± 12.7 | 53.2 ± 10.2 |  | 47.2 ±10.5 | 55.7 ±12.2 | 53.3 ±12.4 |
| Pallidum | 31.3 ± 16.7 |  | 41.4 ± 12.3 | 23.8 ± 28.2 | 42.5 ± 15.4 |  | 33.3 ±10.0 | 24.8 ±29.2 | 31.6 ±15.5 |
| Hippocampus | 60.1 ± 11.6 |  | 46.5 ± 12.1 | 47.8 ± 12.3 | 51.9 ± 14.2 |  | 46.8 ±9.7 | 50.6 ±9.9 | 56.1 ±11.4 |
| WM Lesion | 100.0 ± 0.0 |  | 47.6 ± 15.2 | 35.0 ± 4.0 | 55.4 ± 19.5 |  | 56.3 ±10.5 | 38.5 ±3.0 | 70.9 ±19.4 |
| GM Hyper-perfusion | 78.9 ± 8.6 |  | 55.4 ± 14.4 | 71.4 ± 24.6 | 55.7 ± 12.5 |  | 59.3 ±9.7 | 79.5 ±23.1 | 66.5 ±12.8 |
| GM Hypo-perfusion | 36.6 ± 3.8 |  | 28.9 ± 8.3 | 33.7 ± 11.5 | 31.2 ± 9.3 |  | 31.6 ±5.1 | 38.6 ±11.0 | 34.3 ±6.8 |

**Table S2**. NRMSE (%) of the studied methods with and without motion correction in different regions of the simulated brain phantom.

| ROI |  | No motion correction | | |  | With motion correction | | |
| --- | --- | --- | --- | --- | --- | --- | --- | --- |
|  |  | Standard | 3DLR | MOCHA |  | Standard | 3DLR | MOCHA |
| White matter |  | 49.1 | 57.5 | 48.0 |  | 44.5 | 57.8 | 38.2 |
| Grey matter |  | 65.2 | 52.5 | 56.4 |  | 55.1 | 43.2 | 38.0 |
| Thalamus |  | 55.5 | 49.2 | 43.1 |  | 49.5 | 45.4 | 29.9 |
| Caudate |  | 90.1 | 80.9 | 55.7 |  | 70.6 | 57.6 | 23.9 |
| Putamen |  | 30.0 | 32.6 | 25.2 |  | 29.7 | 25.5 | 18.7 |
| Pallidum |  | 44.8 | 61.7 | 43.9 |  | 34.1 | 60.6 | 23.3 |
| Hippocampus |  | 41.0 | 39.2 | 31.4 |  | 36.7 | 31.3 | 18.9 |
| WM Lesion |  | 109.3 | 184.8 | 83.0 |  | 78.4 | 159.5 | 47.6 |
| GM Hyper-perfusion |  | 52.5 | 34.5 | 48.5 |  | 38.1 | 27.4 | 25.7 |
| GM Hypo-perfusion |  | 37.2 | 33.5 | 31.8 |  | 24.1 | 29.5 | 19.9 |

**Table S3**. The NRMSE performance of MOCHA in different regions of the simulated brain phantom as a function the regularisation parameter $\beta.$

|  |  | Regularization parameter ($\beta)$ | | | | | | |
| --- | --- | --- | --- | --- | --- | --- | --- | --- |
|  | Standard recon. | 1 | 5 | 10 | 15 | 20 | 40 | 100 |
| Grey matter | 48.1 | 47.9 | 44.9 | 44.3 | **44.1** | **44.1** | **44.1** | 44.4 |
| White matter | 67.9 | 84.1 | 64.0 | 58.9 | 56.7 | **55.4** | **53.1** | 51.1 |
| WM Lesion | 41.3 | **32.4** | **32.4** | **33.4** | 34.2 | 35.0 | 37.7 | 43.3 |
| Hyper-perfusion | 27.7 | 28.0 | 23.5 | 22.4 | 22.0 | **21.9** | **21.9** | 22.6 |
| Hypo-perfusion | 20.5 | 33.6 | 23.8 | 21.0 | 19.7 | 18.9 | **17.5** | 16.2 |
| Whole brain | 49.0 | 50.9 | 45.8 | 44.7 | 44.3 | **44.1** | 43.9 | 44.0 |

**Table S4**. Mean and SD of CBF values averaged over 4 healthy subjects.

| ROI |  |  | | |
| --- | --- | --- | --- | --- |
|  |  | Standard | 3DLR | MOCHA |
| White matter |  | 34.5 ± 2.3 | 34.8 ± 4.2 | 26.8 ± 3.1 |
| Grey matter |  | 44.7 ± 3.8 | 44.2 ± 6.7 | 47.1 ± 6.8 |
| Thalamus |  | 36.3 ± 1.9 | 39.2 ± 2.6 | 37.2 ± 4.3 |
| Caudate |  | 29.1 ± 4.1 | 30.8 ± 5.8 | 39.4 ± 12.0 |
| Putamen |  | 33.2 ± 4.3 | 36.5 ± 5.8 | 32.8 ± 7.0 |
| Pallidum |  | 31.4 ± 4.7 | 35.9 ± 5.1 | 29.3 ± 6.2 |
| Hippocampus |  | 35.7 ± 3.4 | 38.1 ± 5.7 | 38.8 ± 7.0 |

**Table S5**. Mean and SD of CBF values averaged over the 2 high-resolution healthy subjects.

| ROI |  | High resolution | Low resolution | |
| --- | --- | --- | --- | --- |
|  |  | Standard | Standard | MOCHA |
| White matter |  | 26.6 ± 14.5 | 28.2 ± 12.6 | 25.7 ± 12.7 |
| Grey matter |  | 40.2 ± 15.7 | 37.7 ± 14.4 | 40.7 ± 14.7 |
| Thalamus |  | 34.3 ± 9.8 | 29.9 ± 9.3 | 32.6 ± 11.8 |
| Caudate |  | 30.6 ± 9.2 | 24.1 ± 8.4 | 28.5 ± 9.1 |
| Putamen |  | 33.0 ± 6.5 | 26.3 ± 5.2 | 27.6 ± 5.7 |
| Pallidum |  | 25.1 ± 7.1 | 20.2 ± 5.8 | 20.1 ± 6.5 |
| Hippocampus |  | 36.1 ± 11.5 | 29.6 ± 8.9 | 33.3 ± 9.5 |

**Table S6**. Mean and SD values of CBF maps for a subject calculated for different control-label pairs.

| Pairs |  | Standard | |  | MOCHA | |
| --- | --- | --- | --- | --- | --- | --- |
|  |  | GM | WM |  | GM | WM |
| 20 |  | 33.6 ± 16.3 | 25.5 ± 12.1 |  | 37.8 ± 19.8 | 20.5 ± 12.1 |
| 10 |  | 35.5 ± 17.2 | 26.7 ± 12.8 |  | 39.7 ± 21.1 | 21.6 ± 13.8 |
| 5 |  | 37.2 ± 18.1 | 27.5 ± 13.9 |  | 42.2 ± 22.4 | 22.5 ± 14.6 |

**Figures**

**
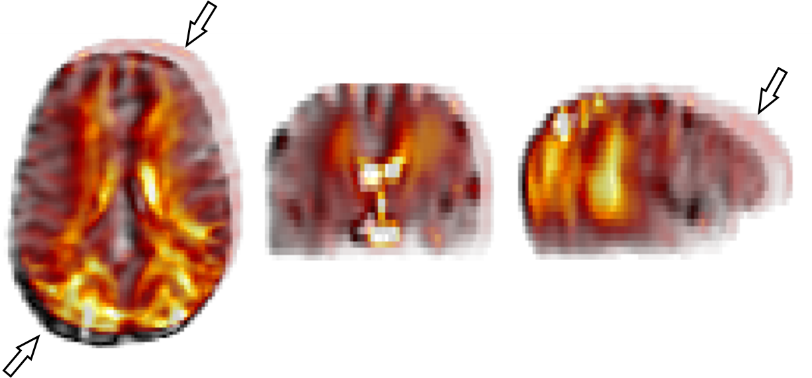
**

**Figure S1.** Simulated motion (translation and rotation) in our simulation dataset.


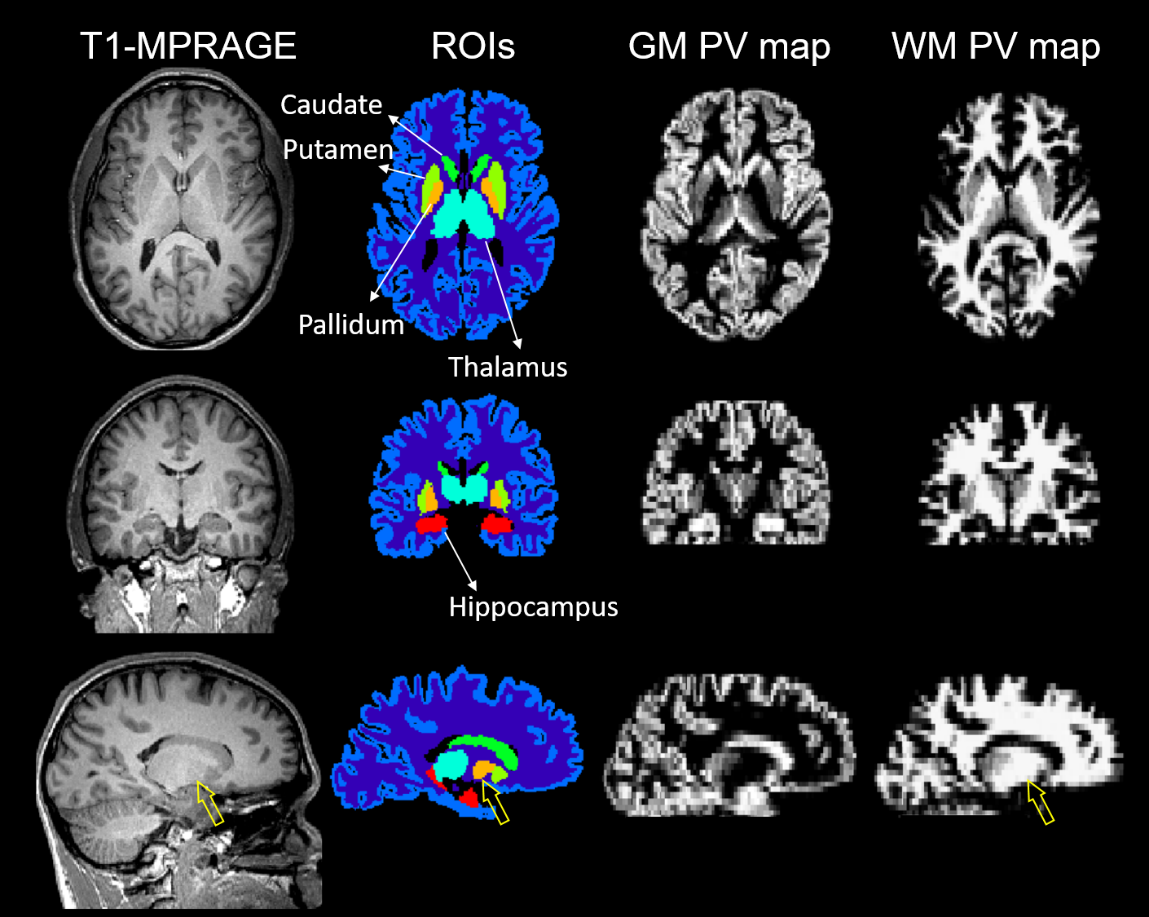


**Figure S2.** The ROIs and GM partial volume estimates obtained from the parcellation of the T1-MPRAGE MR image using the Freesurfer and FSL (FAST and *applywarp*) software. The arrow points to the pallidum that has been erroneously identified as WM.

**Figure S3.** Same as Figure 3, except for the omission of the motion correction step.

**Figure S4.** Same as Figure 3, except for the addition of PSF deblurring for the standard and 3DLR methods. NB: the motion correction step is included for all methods.

|  |  |
| --- | --- |

**Figure S5.** Impact of PSF deblurring on NRMSE performance of the standard and 3DLR methods for simulations.

|  |  |
| --- | --- |

**Figure S6.** CBF profiles of the studied reconstruction methods through simulated WM lesion and GM hyper-perfusion.

**Figure S7.** The NRMSE performance of MOCHA in different regions of the simulated brain phantom as a function of the regularization parameter $\beta$.


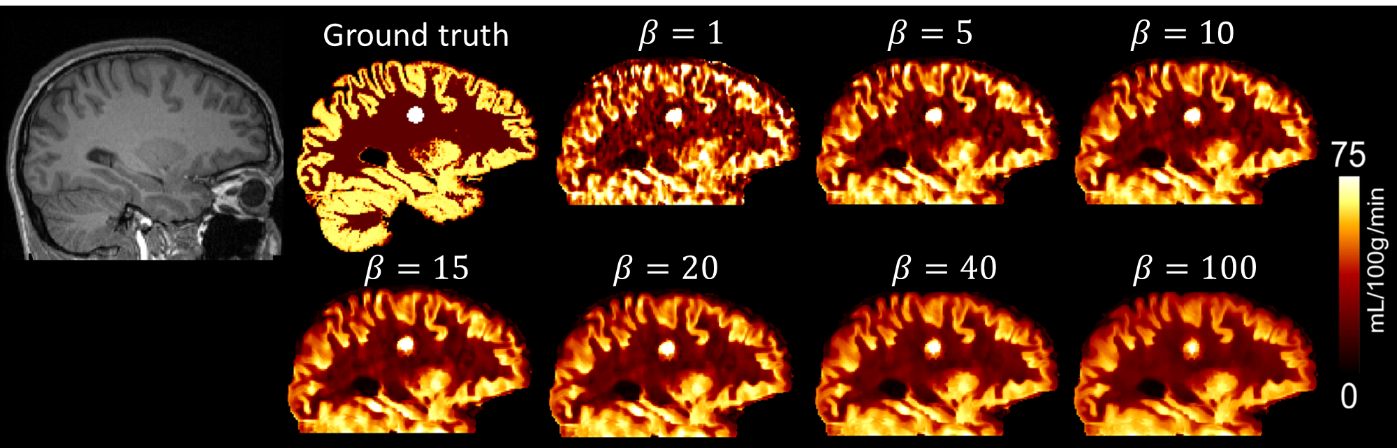


**Figure S8.** The reconstruction results of the MOCHA method as a function of regularisation parameter.

**
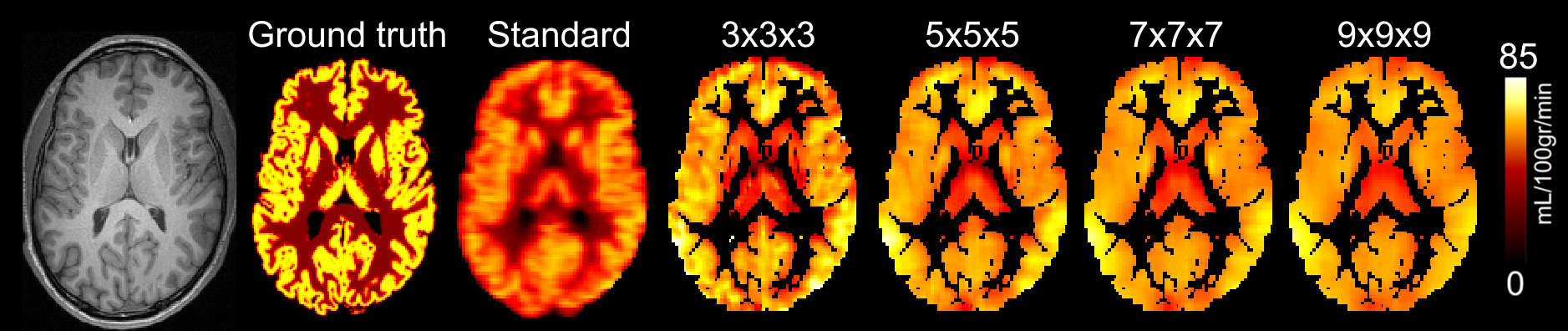
**

**Figure S9.** The impact of kernel size on the qualitative (top) and quantitative (bottom) performance of the 3DLR method in comparison with the standard method and the ground truth simulated brain phantom.


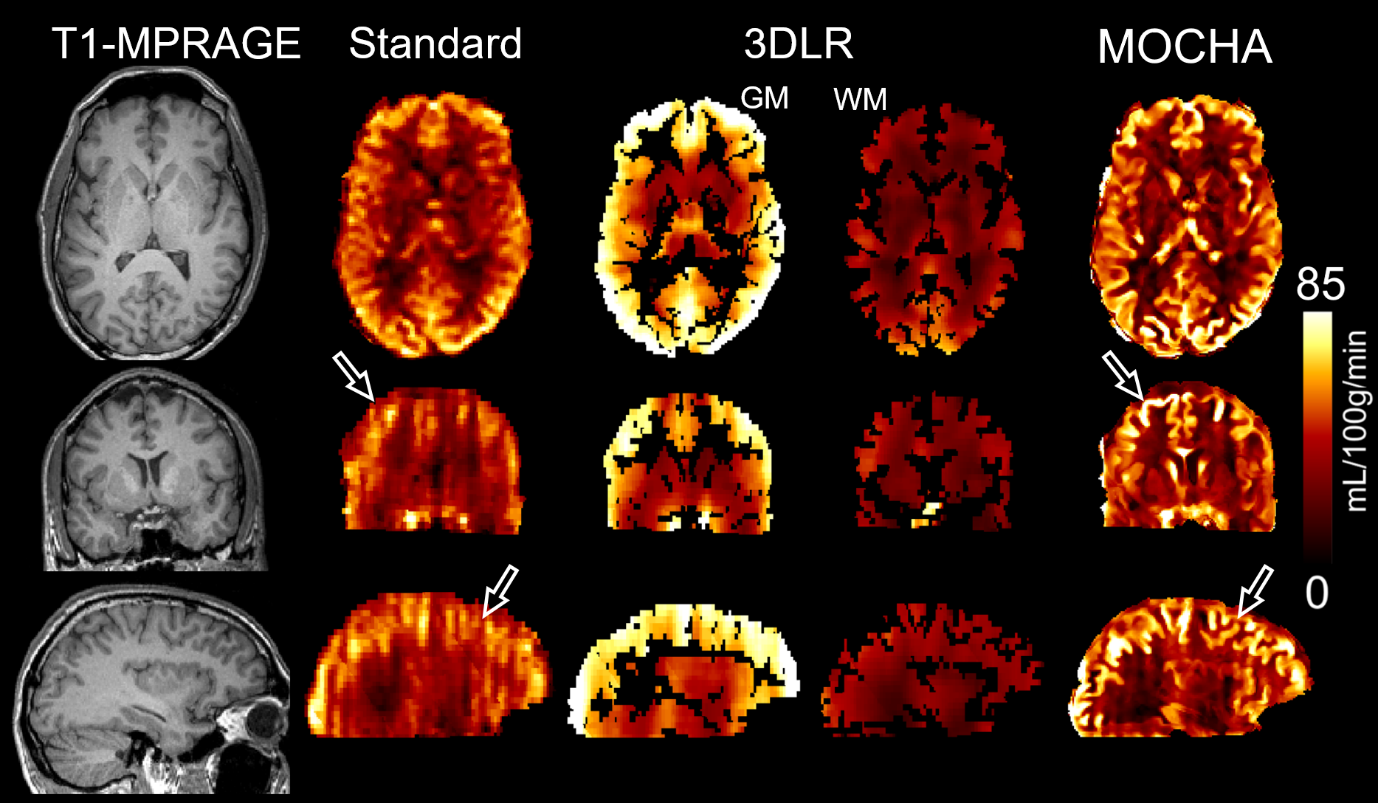


**Figure S10.** CBF results for the subject 3 calculated using the standard, 3DLR and MOCHA reconstruction methods. The arrows point to where there are most notable difference between MOCHA and standard reconstruction methods.

**
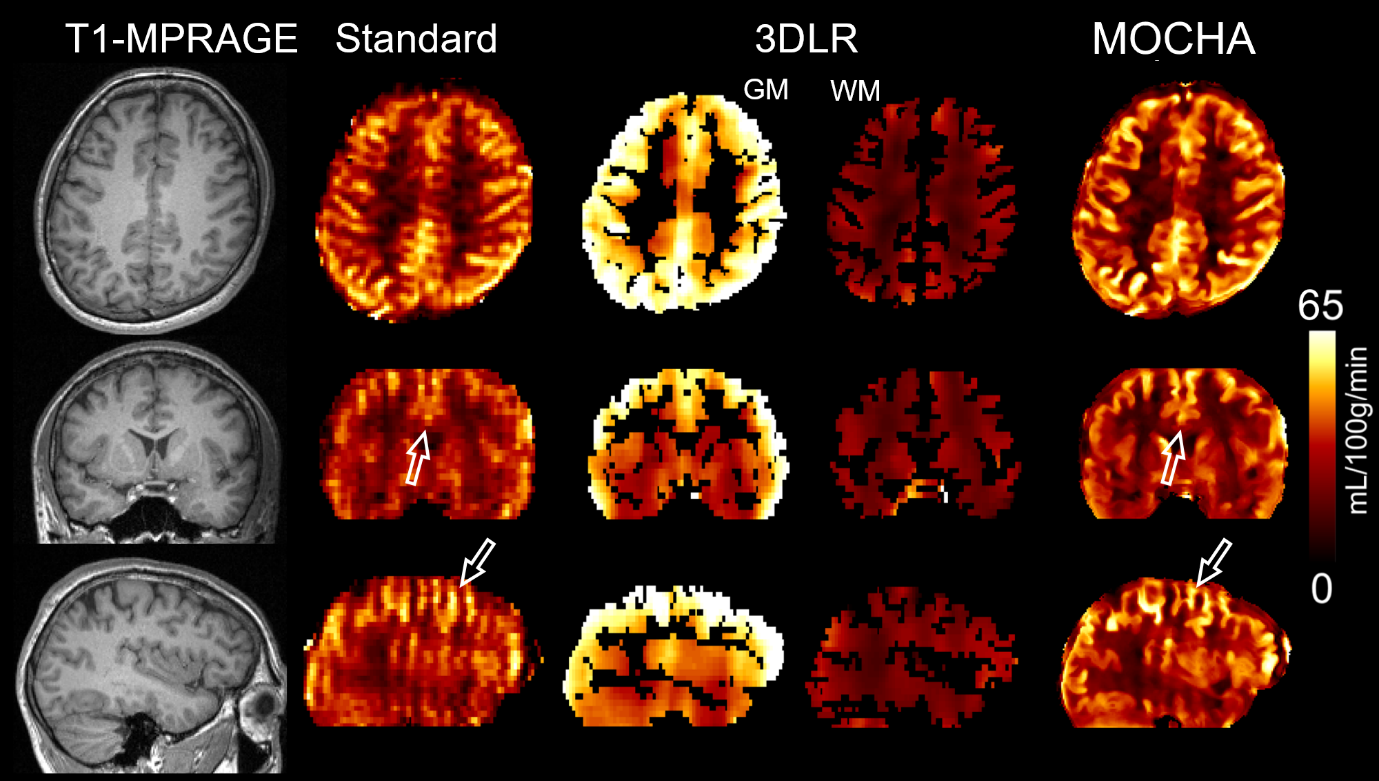
**

**Figure S11.** CBF results for the subject 5 calculated using the standard, 3DLR and MOCHA reconstruction methods. The arrows point to some regions where there are notable difference between MOCHA and standard reconstruction methods.

**Figure S12.** Anatomical image and CBF maps from standard-resolution acquisition (4×4×4 mm^3^; 5 min 40 sec acquisition) standard and MOCHA reconstructions; right) and tripled resolution in the slice direction (‘high-resolution’, 4x4x1.33 mm^3^; 49 min acquisition; standard reconstruction only; left) datasets for subject 6.

**
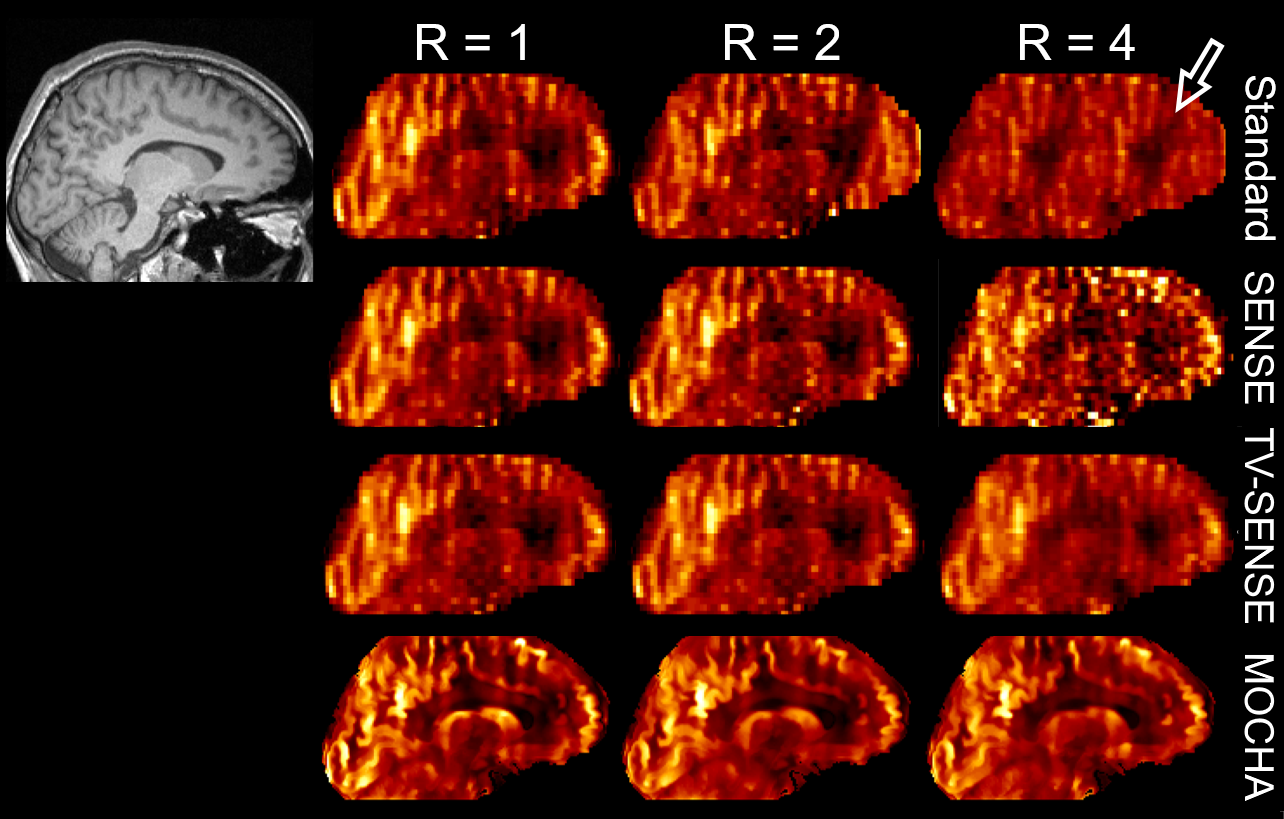
**

**Figure S13.** CBF results of the subject 4 calculated using the standard and MOCHA reconstruction methods for different undersampling factors (R). The arrow shows an undersampling artefact. Standard and SENSE reconstructions show increased noise as R increases. TV-SENSE also shows visible changes between R=2 and R-4. MOCHA shows the highest visual consistency between reconstructions at R=1, R=2 and R=4.


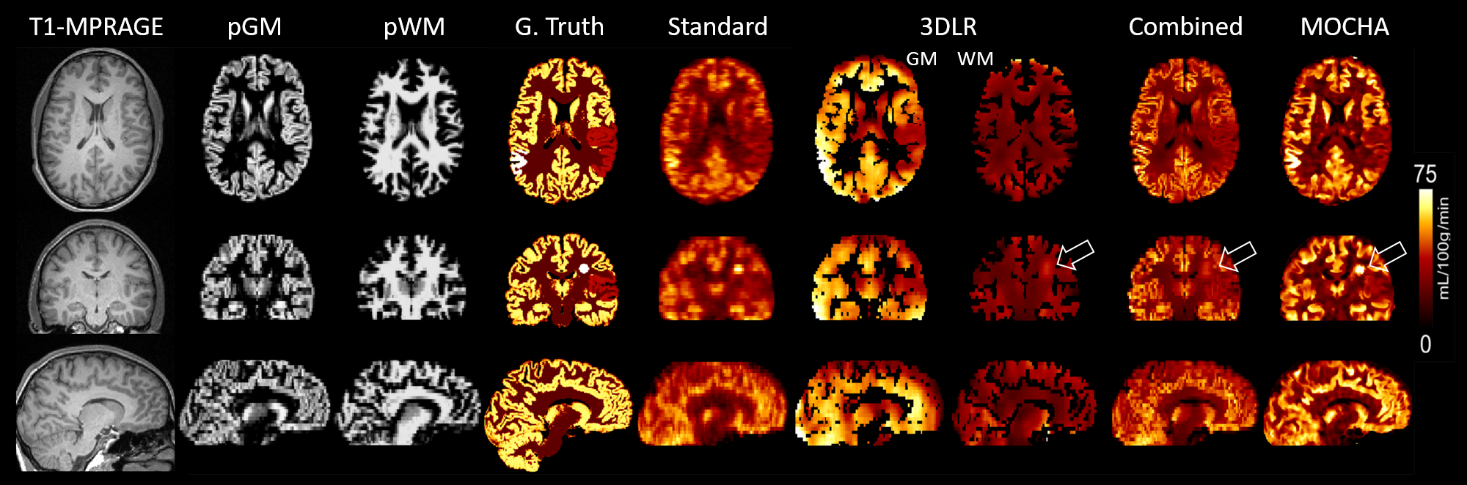

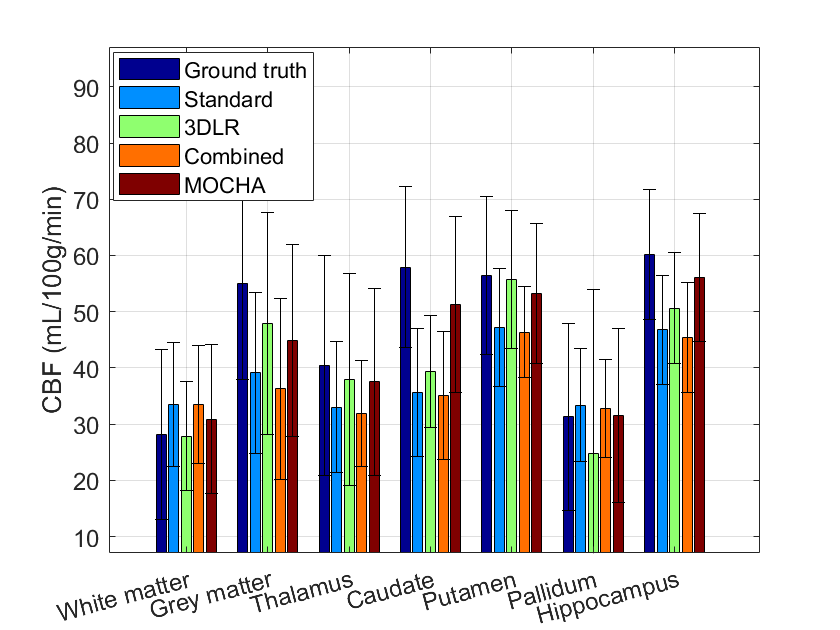


**Figure S14.** Top: similar to Figure 3, including grey and white matter partial volume fractions (pGM and pWM) and a *combined* image equal to pGM* 3DLR_GM + pWM* 3DLR_WM. Bottom: quantitative comparison of the method, as shown, *both 3DLR and MOCHA reduce the discrepancy between the standard reconstruction and the ground truth when CBF is averaged over anatomical regions.*
